# Variable Immunogenic Potential of Wheat: Prospective for Selection of Innocuous Varieties for Celiac Disease Patients via *in vitro* Approach

**DOI:** 10.3389/fimmu.2019.00084

**Published:** 2019-02-04

**Authors:** Jasmine Grover, Parveen Chhuneja, Vandana Midha, Jean Eric Ghia, Dipak Deka, Chandra Shekhar Mukhopadhyay, Neena Sood, Ramit Mahajan, Arshdeep Singh, Ramneek Verma, Ekta Bansal, Ajit Sood

**Affiliations:** ^1^Department of Gastroenterology, Dayanand Medical College, Ludhiana, India; ^2^School of Agriculture Biotechnology, Punjab Agricultural University, Ludhiana, India; ^3^Department of Internal Medicine, Dayanand Medical College, Ludhiana, India; ^4^Section of Gastroenterology, Department of Immunology and Internal Medicine, University of Manitoba, Winnipeg, MB, Canada; ^5^School of Animal Biotechnology, Guru Angad Dev Veterinary and Animal Sciences University, Ludhiana, India; ^6^Department of Pathology, Dayanand Medical College, Ludhiana, India; ^7^Department of Biochemistry, Dayanand Medical College, Ludhiana, India

**Keywords:** celiac disease, immunogenicity, gluten, T-cell epitopes, *in vitro* analysis, cell proliferation, IFN-γ and TNF-α

## Abstract

Celiac Disease (CD) is a multifactorial, autoimmune enteropathy activated by cereal proteins in genetically predisposed individuals carrying HLA DQ2/8 genes. A heterogenous gene combination of the cereal prolamins is documented in different wheat genotypes, which is suggestive of their variable immunogenic potential. In the current study, four wheat varieties (C591, C273, 9D, and K78) identified via *in silico* analysis were analyzed for immunogenicity by measuring T-cell proliferation rate and levels of inflammatory cytokines (Interferon-γ and Tumor Necrosis Factor-α). Peripheral Blood Mononuclear Cells and biopsy derived T-cell lines isolated from four CD patients in complete remission and two controls were stimulated and cultured in the presence of tissue transglutaminase activated pepsin-trypsin (PT) digest of total gliadin extract from test varieties. The immunogenicity was compared with PBW 621, one of the widely cultivated wheat varieties. Phytohaemagglutinin-p was taken as positive control, along with unstimulated cells as negative control. Rate of cell proliferation (0.318, 0.482; 0.369, 0.337), concentration of IFN- γ (107.4, 99.2; 117.9, 99.7 pg/ml), and TNF- α (453.8, 514.2; 463.8, 514.2 pg/ml) was minimum in cultures supplemented with wheat antigen from C273, when compared with other test varieties and unstimulated cells. Significant difference in toxicity levels among different wheat genotypes to stimulate celiac mucosal T-cells and PBMC's was observed; where C273 manifested least immunogenic response amongst the test varieties analyzed.

## Introduction

Celiac disease (CD) is an autoimmune, gluten sensitive enteropathy caused by a complex interplay between genetic and environmental factors, which occur in about 1% of the general population (1). The rising occurrence of the disease is a multifactorial process. Better understanding about the disease, better diagnostics, and higher disease awareness among doctors are some of the reasons. At the same time controlled epidemiological studies have shown true rise in the prevalence (2). One of the hypotheses is changing wheat varieties. Over last few decades, there has been a significant increase in consumption of gluten due to western, wheat- or gluten-rich diet, which is thought to be one of the major causes in the increasing prevalence of CD (3–5).

This is substantiated by the higher prevalence of CD in wheat-consuming states of northern India where disease frequency has gradually mounted (6, 7). According to the reports published in the International Journal of Celiac Disease, the net% increase per year as far as gastrointestinal autoimmune diseases are concerned, was noted to be 6.2. The total net increase of old v/s new surveys of incidence of CD is reported to be 16.8 (8). In parallel, there has been a recent surge in cultivation of high yielding wheat varieties, raising a suspicion whether modern breeding practices may be a contributing factor for development of CD. Modern wheat breeding practices have entirely focused on increasing the yield and disease resistance, without taking into account the possibility of alteration in diversity of some other traits (9). Wheat breeders have developed new varieties without evaluating their impact on the occurrence of CD.

Previous studies have documented that landraces and older wheat varieties contain more diverse gene combinations for prolamins (wheat proteins) in comparison to modern varieties (10, 11). Literature shows variations for specific gene sequences mainly in the epitopic regions of Glia-α9, Glia-α2, Glia-α20, and Glia-α in older landraces (9). In the last decade in the context of CD, the immunogenicity of T-cell specific epitopes has been bought to the forefront (9, 12). The immunogenic potential amongst different hexaploid wheat varieties is variable; hence it is possible that there are breeding-induced differences in the presence and expression of T-cell stimulatory epitopes in modern varieties of wheat (13, 14). This raises the question of, whether there is any specific variety of wheat which is less immunogenic and can be used in breeding programs for developing a wheat genotype completely safe for consumption by patients suffering from CD.

Taken together, recently our group performed a preliminary screening of 43 Indian wheat cultivars represented as pre (1905–1970) and post-green (1971–2011) revolution genotypes (15). Their α-gliadin genes were cloned and sequenced followed by *in silico* analysis and considerable changes were observed within sequence variation in the epitope regions among genes within a variety, and differences between varieties in presence or absence of genes with certain epitopes. We identified and selected four wheat varieties (C591, C273, 9-D, and K78); variant CD epitopes were identified at *Gli-D2* and *Gli-B2* and both intact and variant epitopes at *Gli-A2*, indicating that these epitopes carried reduced load for T-cell stimulatory epitopes (15).

Wheat genome contains 35–150 copies of the α-gliadin genes and sequencing followed by *in silico* studies might not have captured all the variation encompassed in this multigene family. Hence, *in vitro* and *in vivo* analysis was desired to verify the biological activity of the complete proteins. The present study was designed to decipher and to confirm if some wheat varieties are potentially less immunogenic and, therefore, safer for CD patients. We aimed to evaluate the level of immunogenicity of wheat genotypes that emerged from our previous gene sequencing and *in silico* analysis by *in vitro* challenging T-cells and peripheral blood mononuclear cells (PBMC's), isolated from CD patients, against gliadin protein of wheat varieties of our interest.

## Materials and Methods

### Participants and Samples

Prospective study was conducted at Dayanand Medical College and Hospital, Ludhiana, India between January 2015 and February 2017. Four adult patients (≥18 years) diagnosed with CD and on strict gluten free diet (GFD) for ≥2 years, and who have achieved clinical, biochemical and histological remission were considered for recruitment. Diagnosis of CD was established according to the revised European Society for Pediatric Gastroenterology Hepatology and Nutrition (ESPGHAN) criteria (16). Clinical remission was defined as absence of symptoms, biochemical remission as normal anti-tissue transglutaminase IgA antibodies (anti TTG-IgA) and negative anti-endomysial antibodies (anti-EMA). Histological remission was defined as normal villous height/crypt depth (vh/cd) ratio and normal intraepithelial lymphocyte count (IEL count). Written informed consent was obtained in all the cases. Patients in remission for ≤2 years, having active disease (clinical, biochemical or histological symptoms), poor compliance to GFD and significant co-morbidity conditions were excluded. Two healthy controls, asymptomatic for the disease, having normal anti-TTG (IgA), normal histology and negative for Human leukocyte antigen2/8 (HLA DQ 2/8) genes were also enrolled.

For performing T-cell assays 10 ml whole blood was collected in EDTA coated vials. Peripheral blood was also obtained for anti-TTG (IgA) antibodies, anti-EMA antibodies and HLA genotyping. Anti-TTG (IgA) antibodies were measured in the serum by commercially available ELISA kit as per the manufacturer's protocol (Aesku Diagnostics, Cat No. 3508, Germany). Anti-EMA was detected using indirect immunofluorescence (Aesku Diagnostics, Cat No. 512.050, Germany). The subjects underwent gastroduodenoscopy (Olympus GIF-H17 scope, Tokyo, Japan) and multiple biopsies from duodenum were taken during the procedure. One biopsy specimen was sent for histopathological analysis and the rest collected in sterile phosphate buffer saline (PBS) solution supplemented with 100 U/ml Penicillin/Streptomycin (Himedia) for T-cell extraction. HLA-DRB, DQA, and DQB genotyping was determined from peripheral blood DNA using LIFECODES HLA-DRB1; HLA-DQA1/B1 SSO Typing Kits (Immucor Gammma, USA; Cat No. 628923, 628930R) on Luminex-200 Platform (Dr. Lal Path Labs, India). All patient material used (T-cells and PBMC's) was obtained from HLA-DQ2/8^+^ (DRB1^*^03-DQA1^*^05:01-DQB1^*^02:01; DQ2.5) while the controls were negative to both HLA-DQ2/8.

### *In vitro* Gluten Challenge

#### Grain Samples and Gliadin Protein Extraction

A set of four older wheat varieties (C591, C273, K78, and 9D released between years 1934–1974) along with a control variety PBW 621 (released in 2011); currently cultivated and consumed in North Western India, were obtained from Punjab Agricultural University, Ludhiana, India. Gluten protein was extracted from wheat; three extracts were obtained sequentially to extricate whole of the gliadin fraction (17).

**Figure 1:**
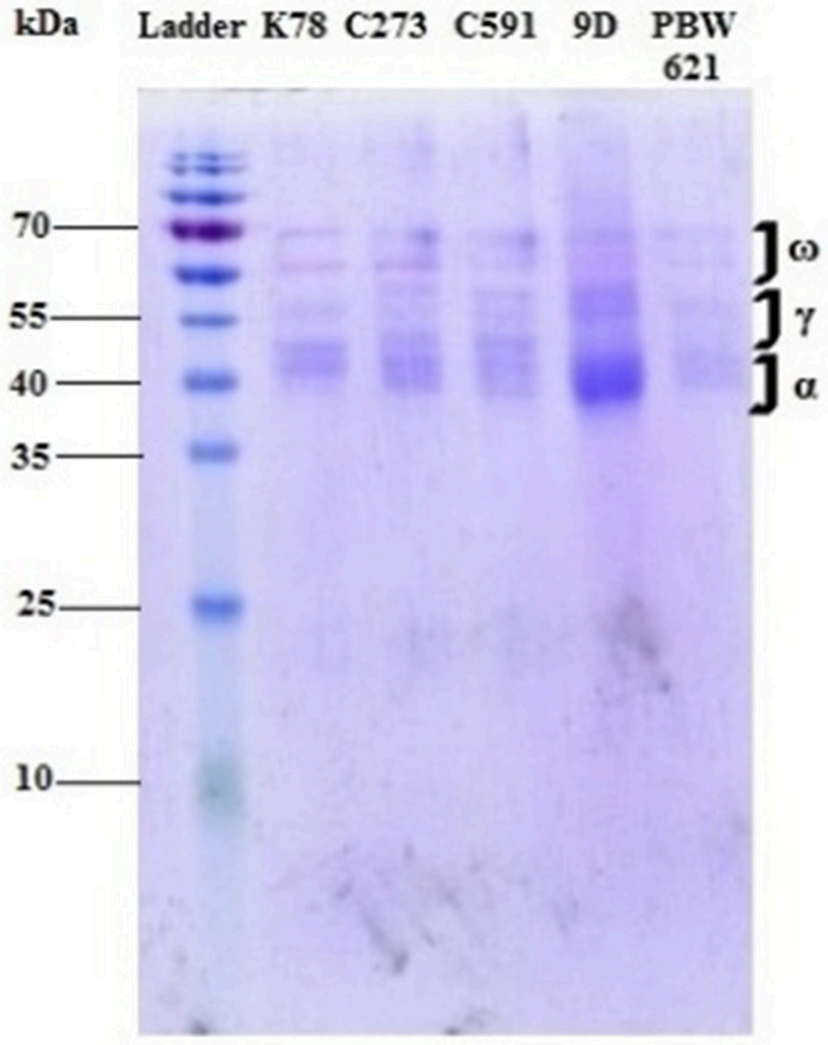
Gliadin profile of different test varieties run on a mini gel at 70 mV for 6 h. Different fractions of gliadin proteins (α, γ, and ω) ranging from 35 to 65 kDa were clearly visible on the gel.

The dissolved protein was dialyzed extensively against 0.01 M acetic acid with three changes of 1 L each, for 24 h at 4°C using a 10 kDa dialysis tubing membrane (ThermoFisher Scientific, USA), lyophilized and completely freeze dried (Christ Freeze drier Alpha 1-2LDplus, Martin Christ Gefriertrocknnungsanlagen GmbH, Germany). Equal amount of gliadin protein at a concentration of 1 mg/ml in PBS solution was loaded on SDS-PAGE gels (15%) on a MINI apparatus (Atto Corporation, Japan) along with a pre-stained ladder (Figure 1, Figure S1) (18). Concentration of protein was estimated using Qubit Assays (ThermoFisher Scientific, USA).

#### Antigen Preparation

PT-digest of 100 mg protein in 0.01 M acetic acid at 1:100 (w/w) ratios was prepared and dialyzed extensively against 0.01 M ammonium bicarbonate and lyophilized (19). Freeze-dried digest was further reconstituted in PBS (pH 7.4), and run through a 45 μm filter. PT-gliadin (1 mg/ml) was incubated with 50 μl of 1 mg/ml guinea pig TTG (Sigma T-5398, Germany) at 37°C for 2 h in RPMI-1640 with 5 mM Calcium Chloride (CaCl_2_) to activate the enzyme to be used in cell cultures (20). Deamidation reaction was quenched by boiling the samples for 5–7 min. at 98°C.

#### Isolation of Biopsy Derived T-Cells and PBMCs

Gliadin fraction from gluten protein of test wheat varieties was analyzed in T-cells derived from duodenal mucosal biopsies and PBMC's from patients with CD in complete remission. The cells were stimulated and cultured in the presence of TTG activated pepsin-trypsin (PT) digest of total gliadin extract from test varieties. Besides this, a recently released variety PBW-621 (2011) and PHA-P (Phytohaemagglutinin-p) were taken as positive control. Isolated T-cells and unfractionated PBMCs were cultured with and without wheat antigen and the response was assessed via measuring cell proliferation rate and cytokine stimulation assays using interferon-γ (IFN-γ) and tumor necrosis factor-α (TNF-α).

**Figure 2:**
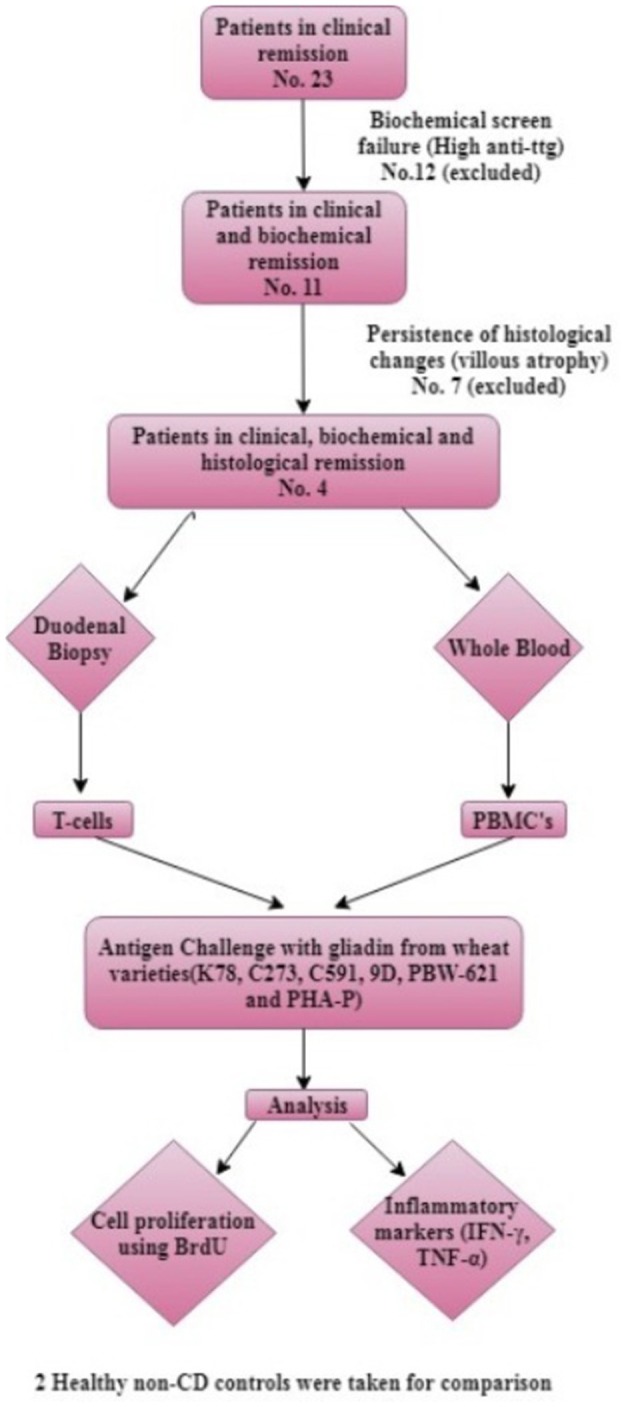
Algorithm of methodology.

For extraction of T-cells each biopsy specimen was finely chopped using a scalpel, digested with Collagenase type 1 (1 mg/ml) (Thermo Fisher Scientific, USA), run through a 70 μm filter, and washed in sterile PBS. The filtrate was centrifuged at 1,000 rpm for 10 min. and resulting cell pellet was dissolved in 1 ml Roswell Park Memorial Institute medium (RPMI-1640) (Himedia, USA) supplemented with 15% fetal bovine serum (FBS) (Gibco, Thermo Fisher Scientific, USA), 2X cocktail of 100 U/ml penicillin/streptomycin (Himedia, USA) and 2.5 mg/ml plasmocin prophylactic (InvivoGen, USA) (complete medium, CM) (19, 21). PBMCs harvested from whole blood were purified from buffy coat using Ficoll-Hisep-1077 (Himedia, USA) gradient centrifugation. Cell number and viability were determined using typan blue. These isolated T-cells and unfractionated PBMCs were exposed to purified gliadin extract from different wheat varieties *in vitro*. For comparison, the whole experiment was replicated in two healthy controls. A schematic representation of the methodology is shown in Figure 2.

#### Bromine Diuridine (BrdU) Proliferation Assay

T-cells extracted from intestinal biopsies were seeded at 5 × 10^5^ cells/well with allogenic PBMC's (10^5^) as antigen presenting cells (APC's) and unfractionated PBMC's at a density of 10^6^ cells/well were placed on a 96-well plate. Both T-cells and unfractionated PBMC's were suspended in T-cell complete media (CM) supplemented with 100 μl deamidated gliadin antigen (1 mg/ml) from respective test wheat varieties, PBW-621 and 2 μl PHA-P (1 mg/ml) (Sigma Aldrich) and cultured at 37°C in 5% CO_2_ for 54 h. In addition, the unstimulated cells (background) cultured in same conditions, acted as negative control. The allogenic PBMC's at a concentration of 1 × 10^5^ cells were cultured separately in same plates to cancel the absorbance for APC's, in order to get the exact proliferation rate of T-cells (19–21). All the wells were incorporated with 10X BrdU labeling reagent (1:10 v/v) for 18 h to label the proliferating cells. Unlabeled cells were taken as BrdU control. After removing supernatant, the cells were washed twice with 200 μl of 0.05% Tween 20 in PBS solution. To remove any traces of media, the plate was micro waved for a minute and the cells were fixed using 70% ethanol and denatured. Blocking buffer (3% bovine serum albumin in PBS) was added to prevent any non-specific binding. BrdU Cell Proliferation Assay was performed according to manufacturer's protocol (BioVision K306-200, USA). Plate was read immediately after addition of stop solution at 450 nm (22).

#### Cytokine Stimulation Assay

Freshly isolated T-cells from intestinal biopsy specimens were resuspended at a concentration of 5 × 10^5^ cells/well with allogenic PBMCs (10^5^) as APC's in 100 μl T-cell CM. PBMCs at a density of 10^6^ cells/well on a 96-well plate were seeded in 100 μl T-cell CM. The cells were stimulated with 100 μl deamidated gliadin antigen (1 mg/ml) of respective test wheat varieties and 2 μl PHA-P (1 mg/ml) (Sigma Aldrich). Cultured cells (both gliadin stimulated and unstimulated) were incubated at 37°C in 5% CO_2_ for 72 h (19–21). Secreted cytokines were harvested from the supernatant by subjecting the plate to centrifugation at 1,500 rpm for 15 min. Cytokine assay was performed using commercial ELISA kits from RayBiotech (Norcross, GA, USA). The cells in culture without protein (background) were taken as negative control, while those supplemented with PBW-621 and PHA-P acted as positive control.

The supernatant was harvested after 72 h of cell cultures and used for cytokine measurement. Both IFN-γ and TNF-α ELISA were performed according to manufacturer's instructions. For each cytokine, standard curves were established to quantify reproducibly the levels of cytokine in culture supernatants (*R*^2^ = 0.9994 for IFN-γ and *R*^2^ = 0.9921 for TNF-α). The assay sensitivity for IFN-γ and TNF-α were 15 and 30 pg/ml respectively.

### Statistical Analysis

Proliferative responses of biopsy derived T-cells and PBMCs to gliadin of test wheat varieties and concentration of IFN-γ and TNF-α from both patients and controls were detected and the data was described in terms of range; mean ± standard deviation (±SD), median, frequencies and relative frequencies as appropriate. Comparison of quantitative variables between the study groups was done using General linear model (Repeated Anova). For comparing categorical data, *post hoc* (least square mean difference-LSD) test to study the interactions between patients and controls as well as between antigens and of antigens with unstimulated cells was performed. A probability value (*p-*value) < 0.05 was considered statistically significant. All statistical calculations were done using Systat 13 version 13.00.55 statistical program for Microsoft Windows.

## Results

### Subjects

A total of 23 patients with CD on strict GFD for ≥2 years were screened, out of which 11 (47.82%) had normal anti-TTG (IgA); but only four patients (17.39%) (25% males; mean age 33.75 ± 11.58 years) had normal endoscopic findings as well as histology. These four patients were enrolled. Out of excluded seven (30.43%) patients, five (21.73%) had evidence of endoscopic disease in the duodenum and two (8.69%) did not achieve histological remission. The control group included two healthy non-CD volunteers (mean age 44.5 ± 21.92 years). The study was approved by Institutional Ethics Committee, Dayanand Medical College and Hospital, Ludhiana, India Demographics and clinical characteristics of the subjects at the time of diagnosis and at the time of inclusion are shown in Table 1.

**Table 1:** Clinical characteristics, TTG antibody levels, histology at diagnosis and inclusion and HLA genotype of study participants.

**Subject**	**Age (years)**	**Weight (Kg)**	**Height (cm)**	**Age at Symptoms onset**	**Age at Diagnosis**	**At the time of diagnosis**			**At the time of inclusion**			**HLA-DQB1**	**Duration on GFD (years)**
						**Anti tTG-IgA (IU/ml)**	**Hb (gm/dl)**	**Marsh Stage**	**Anti tTG-IgA(IU/ml)**	**Hb (gm/dl)**	**Marsh Stage**		
Celiac 1	30–45	62	158	29	35	200	10.3	3C	4	12.4	Zero	02:01[DQ 2 (DQ 2.5)]	5
Celiac 2	18–30	63.1	178	4	6	>300	10.2	3B	7	15.2	Zero	02:01[DQ 2 (DQ 2.5)]	10
Celiac 3	30–45	68.3	168	38	40	>300	11.4	3B	4	13.6	Zero	02:01[DQ 2 (DQ 2.5)]	2
Celiac 4	30–45	72	174	29	29	155	10.6	3B	12	13.7	Zero	02:01[DQ 2 (DQ 2.5)]/02:02 (DQ 2)	8
Control 1	45–60	67	160	NA	NA	NA	NA	NA	2.5	13.8	Zero	Negative	NA
Control 2	18–30	64	168	NA	NA	NA	NA	NA	6	14.6	Zero	Negative	NA

### Proliferation of Biopsy Derived T-Cells and PBMCs

Proliferation of biopsy isolated T-cells and PBMC's was measured by calculating absorbance rate of BrdU labeled cells in CD patients and healthy controls in relation to gliadins from test varieties and other antigens.

**Figure 3:**
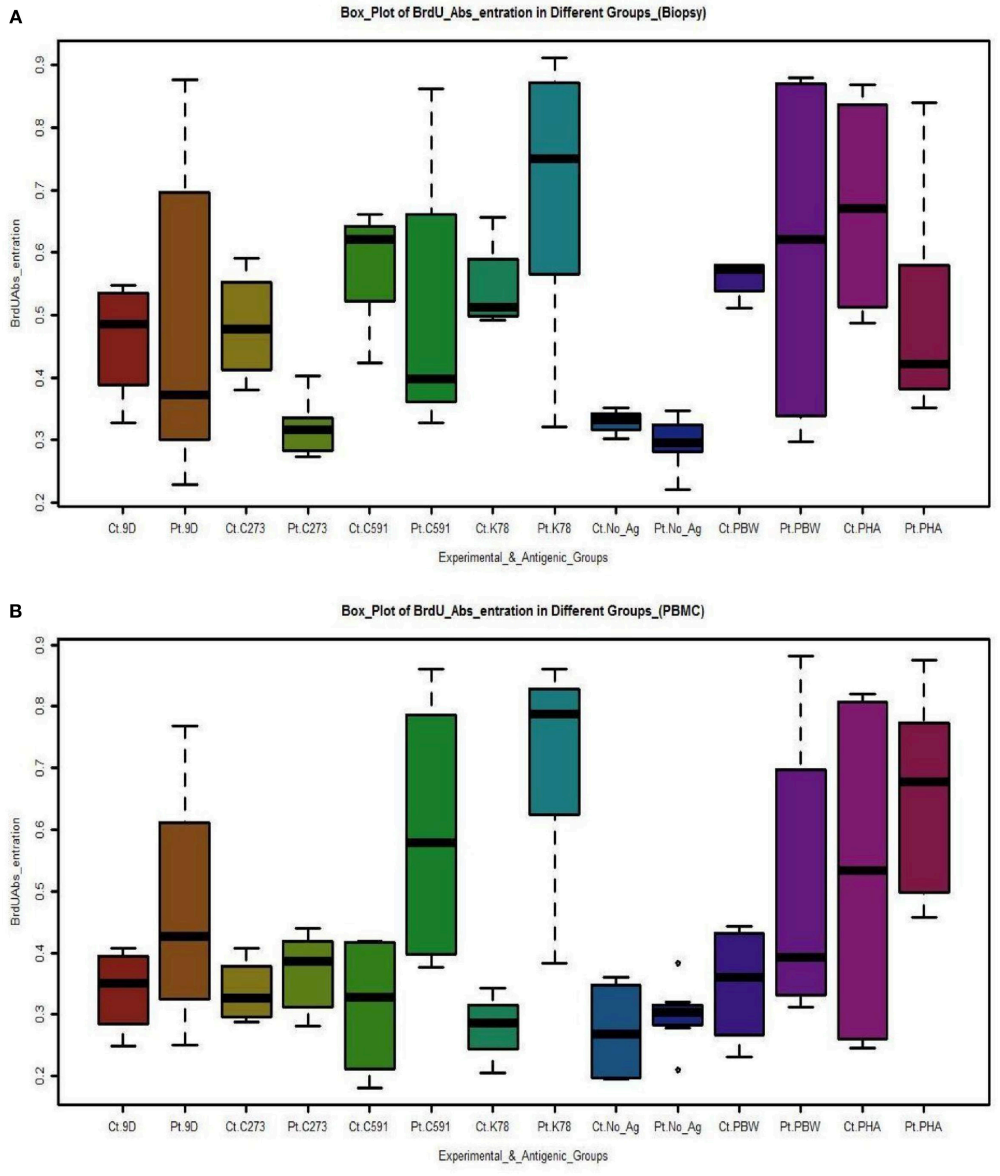
Boxplots depicts the proliferation of **(A)** Biopsy derived T-cells and PBMCs **(B)** measured by absorbance rate of BrdU labeled cells in CD patients and controls in correlation to gliadins from test varieties and other antigens. Cultures supplemented with gliadin from variety C273 exhibited least activation being almost equal to background. [Pt, Patients; Ct, Controls; No_Ag, No-Antigen)]. The box-plots were drawn by R programming language (R version 3.4.2). Upper and lower box plot margins represent the interquartile range; middle bar indicates the median. The points outside the ends of the whiskers are outliers. The graph was plotted based on the absolute number of cells stained with BrdU on each area measured.

Variable T-cell proliferation in both duodenal biopsy derived T-cells and PBMCs was observed in response to different antigens in CD patients and control group. Maximum proliferation was observed in patient T-cells cultured with antigen derived from wheat variety K78 (0.701 ± 0.218) and minimum in those administered C273 antigen (0.318 ± 0.043). No significant difference was determined in T-cell cultures of non-celiac controls supplemented with gliadin from K78 (0.544 ± 0.076) and C273 (0.482 ± 0.091) (Table 2). In parallel, the highest proliferation of PBMC cultures was also seen in those supplemented with gliadin from wheat genotype K78 (0.715 ± 0.175) and the lowest in C273 genotype (0.369 ± 0.060) (Table 2). On the contrary, proliferation rate in PBMC's of non-celiac controls cultured with or without wheat antigen was similar irrespective of the wheat variety, but were higher with PHA-P (0.534 ± 0.310) (Table 2). Figures 3A,B exhibits the range and mean proliferation values of T-cells and unfractionated PBMCs in both CD patients and non-celiac controls when cultured with wheat antigen from different test varieties and PHA-P.

**Table 2:** Mean proliferation rates of Biopsy derived T-cells and PBMCs in treated CD patients and non-CD controls when cultured for 72 h with wheat antigen from different test varieties.

**Least squares means**				
	**Biopsy derived T-cells**		**PBMCs**	
**Antigens**	**LS Mean ± SD (Patients)**	**LS Mean ± SD (Controls)**	**LS Mean ± SD (Patients)**	**LS Mean ± SD (Controls)**
K78	0.701 ± 0.218	0.544 ± 0.076	0.715 ± 0.175	0.280 ± 0.057
C273	0.318 ± 0.043	0.482 ± 0.091	0.369 ± 0.06	0.337 ± 0.054
C591	0.503 ± 0.222	0.582 ± 0.108	0.595 ± 0.212	0.314 ± 0.122
9D	0.481 ± 0.256	0.462 ± 0.098	0.469 ± 0.193	0.340 ± 0.071
PBW-621	0.605 ± 0.276	0.560 ± 0.033	0.504 ± 0.227	0.348 ± 0.100
PHA-P	0.495 ± 0.178	0.675 ± 0.190	0.654 ± 0.158	0.534 ± 0.316
No-Ag	0.296 ± 0.039	0.329 ± 0.020	0.300 ± 0.048	0.273 ± 0.087

In addition, the proliferative interactions of patient T-cell cultures supplemented with wheat antigen from variety C273, showed significant differences with K-78 (*p* = 0.000), C-591 (*p* = 0.037), PBW-621 (*p* = 0.002), and PHA-P (*p* = 0.047). Similar results were seen with PBMC cultures.

### Cytokine Assays

Specific cytokine production in response to gliadins from concerned wheat varieties, PBW621, PHA-P, and unstimulated cells was also calculated.

#### Detection of IFN-γ Responses to Gliadins/PHA-P

**Figure 4:**
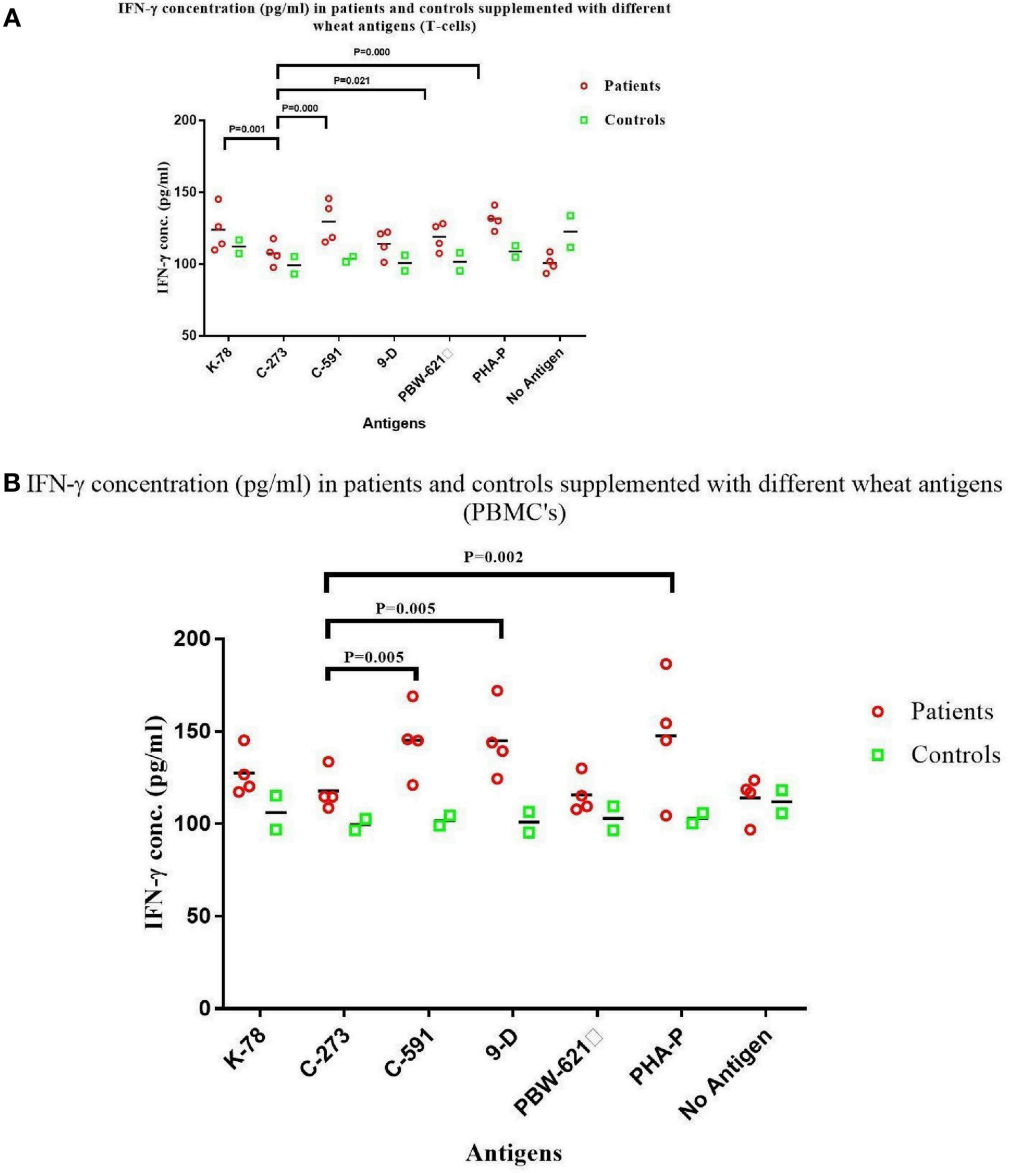
IFN-γ levels (pg/ml) in the supernatant of non-stimulated and stimulated cultures with gliadin protein from test varieties, and PHA-P in Celiac disease patients and Non-Celiac controls in **(A)** Duodenal Biopsy derived T-cells and **(B)** PBMCs. The graphs were constructed using GRAPHPAD PRISM 7 software.

The mean (±SD) *in vitro* production of IFN-γ after antigenic stimulation for 72 h was measured and compared in both the experimental groups [CD patients and non-CD controls (Figures 4A,B)]. All the samples in experimental group produced IFN-γ in response to gliadins from test wheat varieties and PHA-P, although with a variable intensity. The interaction between different wheat antigens with both experimental groups was significant in T-cells (*p* = 0.000), as well as PBMC's (*p* = 0.043). Interestingly, a weak IFN*-*γ response was detected in patient T-cell cultures stimulated with C273 gliadin (107.4 ± 7.8 pg/ml), as compared to the responses with wheat antigen from C591 (129.6 ± 14.4 pg/ml). Similarly, IFN-γ secretion in supernatant of PBMC's from patients cultured with C273 gliadin (117.9 ± 12.4 pg/ml) was the least (Table 3, Table S2). Higher levels of IFN-γ were noticed in supernatants of patient derived T-cell cultures supplemented with K78 (*P* = 0.001), C 591 (*P* = 0.000), PBW 621(*P* = 0.021), and PHA-P (*P* = 0.000) in contrast to those stimulated with gliadin from variety C273 (Figure 4A). However, when T-cells and PBMC's of non-CD control cultures were analyzed for IFN-γ levels, nearly similar secretions were measured irrespective of the wheat antigen (Table 3, Table S2).

**Table 3:** Mean *IFN-*γ and TNF-α secretion in Biopsy derived T-cells and PBMCs in treated CD patients and non-CD controls when cultured for 72 h with wheat antigen from different test varieties.

**Least squares means**								
**Antigens**	**IFN-γ**				**TNF-α**			
	**Biopsy derived T-cells**		**PBMC's**		**Biopsy derived T-cells**		**PBMC's**	
	**LS Mean ± SD (Patients)**	**LS Mean ± SD (Controls)**	**LS Mean ± SD (Patients)**	**LS Mean ± SD (Controls)**	**LS Mean ± SD (Patients)**	**LS Mean ± SD (Controls)**	**LS Mean ± SD (Patients)**	**LS Mean ± SD (Controls)**
K-78	123.43 ± 15.16	112.20± 5.70	127.43 ± 11.64	106.16 ± 10.67	522.81 ± 14.07	508.00 ± 72.45	545.37 ± 19.65	508.00 ± 72.45
C-273	107.43 ± 7.84	99.25 ± 5.70	117.93 ± 12.47	99.70 ± 4.16	453.81 ± 6.75	514.25 ± 62.19	463.81 ± 8.79	514.25 ± 62.19
C-591	129.64 ± 14.44	103.45 ± 7.23	145.29 ± 21.05	101.79 ± 3.87	511.50 ± 14.82	542.37 ± 48.36	548.93 ± 38.00	542.37 ± 48.36
9-D	114.20 ± 9.35	100.75 ± 7.25	145.02 ± 29.07	100.95 ± 7.11	531.18 ± 17.00	514.62 ± 26.24	539.93 ± 39.22	514.62 ± 26.24
PBW-621	119.10 ± 16.37	101.67 ± 9.41	115.68 ± 16.91	103.04 ± 8.37	535.75 ± 40.32	537.37 ± 10.07	523.56 ± 38.80	537.37 ± 10.07
PHA-P	131.54 ± 8.02	108.87 ± 4.58	147.72 ± 34.94	103.08 ± 3.57	484.25 ± 60.18	481.75 ± 7.50	470.75 ± 18.28	481.75 ± 7.50
No-Ag	100.75 ± 5.89	122.62 ± 14.86	114.10 ± 15.20	112.00 ± 10.65	448.37 ± 6.27	476.12 ± 19.51	452.68 ± 8.90	476.12 ± 19.51

It was also observed that the concentration of IFN-γ from patient derived cultures activated with wheat antigen from C273 were comparable with those of unstimulated cells (Figures 4A,B). However, for unstimulated cells, IFN-γ production in biopsy derived T-cell cultures was more in controls as compared to the patients (Table 3, Table S2). The higher secretion of IFN-γ was measured in both patient derived T-cells and PBMC cultures given antigenic stimulation by wheat genotype C591 indicating a strong immunogenic potential of this variety to prompt CD.

#### Detection of TNF-α in Stimulated and Unstimulated Cultures

**Figure 5:**
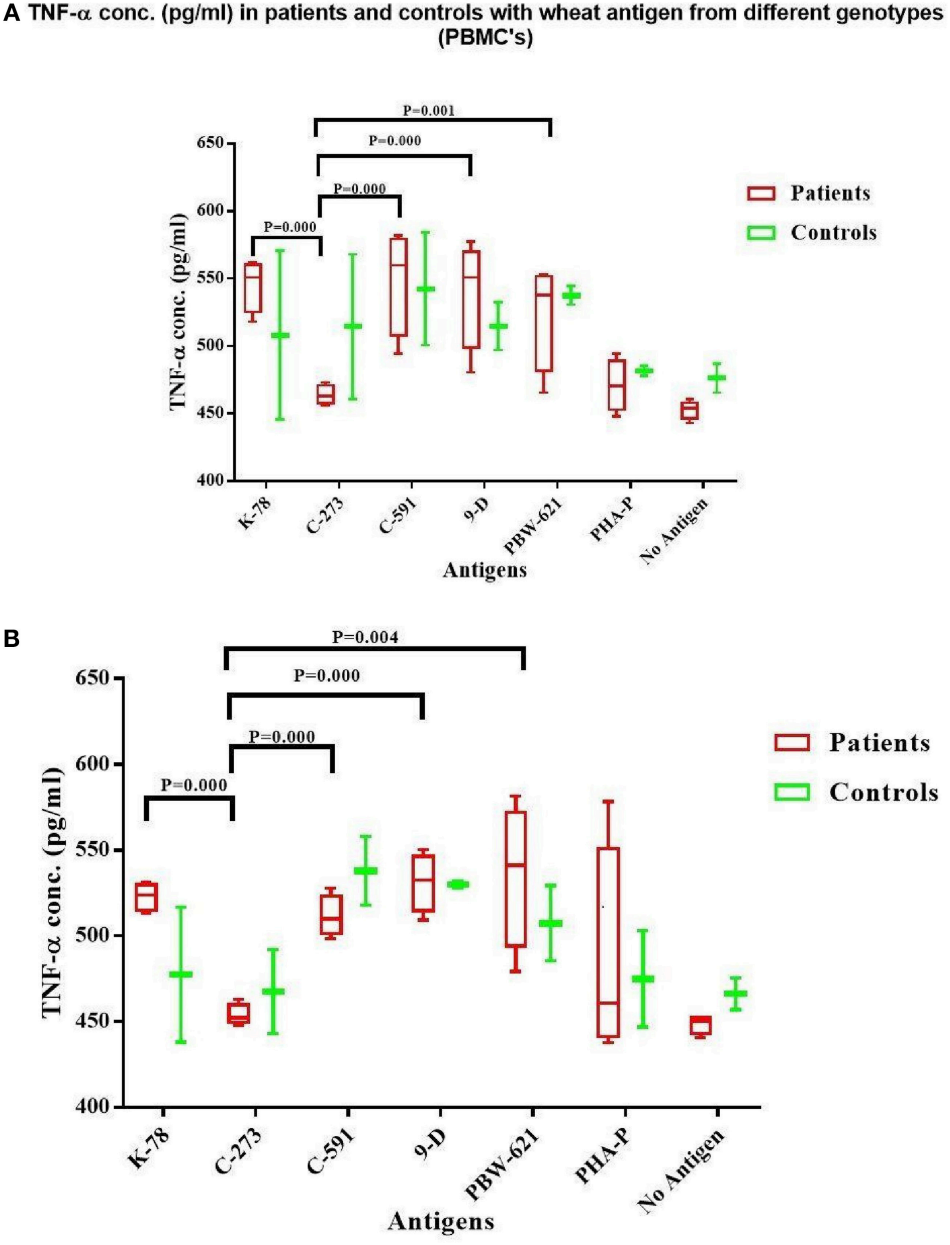
*TNF-*α level (pg/ml) in the supernatant of non-stimulated and stimulated cultures with gliadin protein from test varieties, and PHA-P in Celiac disease patients and Non-Celiac controls in **(A)** PBMCs and **(B)** Duodenal Biopsy derived T-cells. The graph was constructed using GRAPHPAD PRISM 7 software.

Supernatant harvested after culturing the cells for 72 h. was also used to detect *TNF-*α production. The levels of TNF-α production in gliadin stimulated PBMC cultures in treated celiac patients (range = 463.8–548.9 pg/ml) and controls (range = 508.0–542.3 pg/ml) showed no significant differences (Figure 5A). Concentration of TNF-α were similar in PBMC cultures prepulsed with gliadin from K78, C591, 9D, and PBW-621, thus showing a non-significant trend (*P* = 0.052) (Table S3). T-cell cultures reported a concordant pattern with minor variations within the subjects (range = 453.8–535.7 pg/ml for patients; 514.2–542.3 pg/ml for controls) (*P* = 0.580) (Figure 5B). As with IFN-γ, TNF-α concentration was also minimal in patient cultures supplemented with C273 gliadin (T-cells = 453.8 pg/ml; PBMC's = 463.8 pg/ml) which was, however, non-significantly different from background (*P* = 0.499; *P* = 0.501, respectively).

A higher concentration of TNF-α was observed in both treated CD patients and controls, activated with gliadins from K78, C591, 9D, and PBW-621 in parallel to C273 (Table 3, Table S2).

Table 4 gives a schematic layout of the immunogenicity levels in different test varieties of wheat in both CD patients and non-CD healthy controls.

**Table 4:** Schematic layout of Immunogenicity levels of test wheat varieties in celiac disease patients and Non-celiac healthy controls.

**S.No**.	**Antigen type**	**Antigen**	**Experimental groups**	**Immunogenicity level**					
				**Tissue type**					
				**D2 Biopsy**			**Blood**		
				**Cell proliferation**	**Inflammatory cytokines**		**Cell proliferation**	**Inflammatory cytokines**	
					**IFN-γ**	**TNF-α**		**IFN-γ**	**TNF-α**
1	Test varieties	K 78	Patients	+ + + +	+ +	+ + +	+ + + +	+	+ + + +
			Controls	+ +	+	+ +	–	–	+ +
2		C 273	Patients	–	–	–	–	–	–
			Controls	+	–	+ + +	–	–	+ + +
3		C 591	Patients	+ +	+ + +	+ + +	+ +	+ + +	+ + + +
			Controls	+ +	–	+ + + +	–	–	+ + + +
4		9 D	Patients	+	+	+ + +	+	+ + +	+ + + +
			Controls	+	–	+ + +	–	–	+ + +
5	Positive control (wheat)	PBW 621	Patients	+ + +	+ +	+ + + +	+ +	–	+ + +
			Controls	+ +	–	+ + + +	–	–	+ + + +
6	Positive control (mitogen)	PHA-P	Patients	+	+ + +	+	+ + +	+ + + +	+
			Controls	+ + +	+	+	+ +	–	+
7	Negative control	No Antigen	Patients	–	–	–	+ +	–	–
			Controls	–	+ +	–	–	–	+

*[Immunogenicity levels—Highest (+ + + +), Higher (+ + +), High (+ +), Low (+), Non-immunogenic (–)]*.

## Discussion

In the current pilot study, the immune toxicity of wheat antigen from four genotypes of old hexaploid wheats, C591, C273, 9D, and K78 and the current wheat variety PBW-621was assessed using *in vitro* assays on CD patient-derived gliadin reactive T-cells and PBMCs. These specific varieties were selected from our previous study, where based on *in silico* and gene sequencing analysis we reported these to carry a reduced load for T-cells stimulatory epitopes (15). Our findings show that a substantial immunogenic difference exists among these four varieties of wheat. The results predicted that total gliadin extract from four test varieties K78, 9D, C591, C273, and PBW621 manifested differential ability to stimulate celiac mucosal T-cell lines. This was specified by cell proliferation assays and IFN-γ and TNF-α production. Gliadin extract from an old variety C273 released by the Department of Agriculture, Punjab, India in 1957 showed least immunogenicity amongst all test varieties analyzed. Rate of multiplication of patient derived T-cells and PBMCs was observed to be minimal in cultures stimulated with C273 gliadin. On the contrary an increased proliferation was shown with other varieties K78, C591, and PBW621 (control) (Figures 3A,B, Table 2). Similarly, secretion of IFN-γ and TNF-α were least in C273 activated cultures (Table 3, Figures 4, 5). These findings on comparison with other wheat genotypes taken (K78, C591, 9D, and PBW621), with controls and with unstimulated cultures were consistent with low immunogenic potential of C273. We feel that proliferation assay from both gluten reactive T-cell and PBMCs when combined with production of inflammatory cytokines, should detect the potential of gliadin peptides to activate celiac lesion T-cells.

The identification of less/non-immunogenic wheat species is an important milestone that could help patients or even prevent CD. One of the major restricting factors, however, is the lack of an operative animal model that could fully correlate with the disease pathogenesis; hence *in vitro* models have been developed. Staining with mAbs limits the revelation of all present T-cell epitopes due to its shorter epitope recognition site, and due to interference of some other sites that do not represent complete epitopes (9). Therefore, intestinal T-cell clones are considered as sensitive and accurate monitors to analyze complex protein digests from different wheat accessions (13, 23). A potential pitfall is that direct *in vivo* gluten exposure causes destruction of villous architecture along with inducing T-cell activation (24). This relationship cannot be formally established *ex vivo* but we believe that assessing gluten response of celiac derived T-cells is a prime *in vitro* marker to explore the toxicity of any wheat variety. To minimize this, polyclonal T-cells from four different treated CD-patients and two non-CD healthy controls for complimentary testing were taken.

A higher degree of polymorphism exists in gluten genes in individual wheat accessions, which may be suggestive of distinct toxicity profile of different wheat genotypes (12). This has prompted analysis of selected old hexaploid wheat genotypes to find allelic variants in modern bread wheats which are purported to be significant in CD. Previous studies have failed to identify non-toxic wheat. However, as the knowledge on T-cells stimulatory epitopes is increasing interesting observations are being reported (9, 25–28). Both the amount of gluten in the wheat genotypes along with the level of toxic T-cell stimulating epitopes contribute toward the noxious nature of wheat for CD patients (29, 30). In 2005, Molberg et al. defined highly immunodominant and immunostimulatory αG-33 mer fragment encoded by genes located on Gli-2 locus of chromosome 6D (23). Experimental data from different *Triticum* species with AA, AABB and AABBDD genotypes reinforced the cytotoxicity of wheat varieties against intestinal epithelial cells, however; in contrast the studies conducted on CaCO-2/TC7 and K562(S) cells reported no cytotoxicity (28, 31). Another study documented emmer and durum wheats to exhibit lower reactivity than common wheat due to lack of D-genome (32). But even these species have been shown to express T-cell immunogenic α- and γ-epitopes, their reactivity profile is not universal, therefore, safety of wheat species depends on an individual's response to gluten (29, 33). Previous studies comparing European heritage and modern wheats, identified less toxic wheat landraces (9). Despite a seemingly high prevalence of CD in India (1.04%) (34), only one report from our group; has been published so far to evaluate Indian wheat cultivars (15). This trial is an important step in the direction where old Indian wheat varieties have been examined for their potential to elicit CD.

Pathogenesis of CD asserts its direct effect on the epithelium via activation of CD4^+^ T-Cells in lamina propria by processed gluten peptides (35). In the past decade, central role of adaptive immunity to cause tissue damage in CD has been highlighted. The cascade involves activating cytotoxic T-cells and secreting pro-inflammatory cytokines that triggers enterocyte apoptosis ^[33]^. Studies using prolamins of wheat species from *Triticum* have assessed the toxic effects on CaCO-2/TC7 and K562(S) cells, measuring cell viability, Nitric oxide (NO) release and transepithelial resistance (TER) (9) while others have screened the varieties using monoclonal antibody staining technique (28). Gluten specific T-cell lines have also been generated from celiac biopsy samples measuring the toxicity of ancestral wheat species by either performing T-cell assays (13, 23) or measuring secretions of IFN-γ (20, 36, 37) and TNF-α (38) production. Our current study has shown that selected wheat varieties (K78, C591, 9D, and PBW621) activate celiac specific T-cells by cell proliferation and increased production of cytokines.

C273 is a pre-green revolution variety which is medium tall and low yielding. Hence, it failed to become a popular choice among cultivators (39, 40). C273 along with other C series varieties, however, are reported to be high quality wheats especially for flat bread (*chapatti*) purposes. The increasing prevalence of CD worldwide is a concern for both the consumers and health professionals. There is no doubt that improved and convenient diagnostics and hygiene hypothesis have contributed to this increase. But owing to extensive breeding of wheat in past 50–100 years, genes coding for gluten proteins may have lost their heterogeneity (41, 42). Kaur et al. (15, 40) and the present study emphasize the testing of old wheat accessions.

In conclusion, by considering the level of toxic T-cell epitopes our data indicate that with the use of gluten-specific T-cells and PBMCs, wheat genotypes containing minimally harmful gluten sequences can be selected. Our results based on *in vitro* analysis, exposing duodenal mucosal biopsy derived T-cells extracted from CD patients in remission to different wheat varieties reveal C273 genotype as a potential safer variety, but a larger study should be conducted to confirm the finding.

## Ethics Statement

Ethics Committee: Institutional Ethics Committee, Dayanand Medical College and Hospital, Ludhiana, India. All the patients were informed about the pros and cons of the study and procedures that were to be used and a consent was obtained from all the ones who agreed.

## Author Contributions

AjS and JG: conception and design, collection, analysis and interpretation of the data, drafting of the article, critical revision of the article for important intellectual content, final approval of the article; PC and VM: conception and design, critical revision of the article for important intellectual content, final approval of the article; JEG, DD, NS, RM, RV, and EB: critical revision of the article for important intellectual content, final approval of the article; CM: statistical analysis, critical revision of the article for important intellectual content, final approval of the article; ArS: drafting of the article, critical revision of the article for important intellectual content, final approval of the article.

### Conflict of Interest Statement

The authors declare that the research was conducted in the absence of any commercial or financial relationships that could be construed as a potential conflict of interest.
