# Supplementary material for: Variable Immunogenic Potential of Wheat: Prospective for Selection of Innocuous Varieties for Celiac Disease Patients via in vitro Approach
Source: Front Immunol. 2019 Feb 4;10:84. doi: 10.3389/fimmu.2019.00084 (PMC6371638; doi:10.3389/fimmu.2019.00084)
Supplement: Supplementary file 1 [file Data_Sheet_1.docx]

**Supplement Material**

1. BRDU Cell proliferation (Biopsy)

| **Fisher's Least-Significant-Difference Test** | | | | | |
| --- | --- | --- | --- | --- | --- |
| **ANTIGEN(i)*EXP_- GR(i)** | **ANTIGEN(j)*EXP_- GR(j)** | **Difference** | **p-Value** | **95% Confidence Interval** | |
|  |  |  |  | **Lower** | **Upper** |
| K78*Patients | K78*Controls | 0.157 | 0.146 | -0.056 | 0.371 |
| K78*Patients | C273*Patients | 0.383 | 0.000 | 0.209 | 0.557 |
| K78*Patients | C273*Controls | 0.219 | 0.044 | 0.006 | 0.432 |
| K78*Patients | C591*Patients | 0.198 | 0.027 | 0.023 | 0.372 |
| K78*Patients | C591*Controls | 0.119 | 0.270 | -0.094 | 0.332 |
| K78*Patients | 9D*Patients | 0.221 | 0.014 | 0.046 | 0.395 |
| K78*Patients | 9D*Controls | 0.240 | 0.028 | 0.026 | 0.453 |
| K78*Patients | PBW621*Patients | 0.096 | 0.275 | -0.078 | 0.270 |
| K78*Patients | PBW621*Controls | 0.142 | 0.190 | -0.072 | 0.355 |
| K78*Patients | PHA-P*Patients | 0.206 | 0.021 | 0.032 | 0.380 |
| K78*Patients | PHA-P*Controls | 0.027 | 0.805 | -0.187 | 0.240 |
| K78*Patients | No Ag*Patients | 0.405 | 0.000 | 0.230 | 0.579 |
| K78*Patients | No Ag*Controls | 0.372 | 0.001 | 0.158 | 0.585 |
| K78*Controls | C273*Patients | 0.226 | 0.039 | 0.012 | 0.439 |
| K78*Controls | C273*Controls | 0.062 | 0.619 | -0.185 | 0.308 |
| K78*Controls | C591*Patients | 0.040 | 0.707 | -0.173 | 0.254 |
| K78*Controls | C591*Controls | -0.038 | 0.758 | -0.285 | 0.208 |
| K78*Controls | 9D*Patients | 0.063 | 0.556 | -0.150 | 0.277 |
| K78*Controls | 9D*Controls | 0.082 | 0.508 | -0.164 | 0.329 |
| K78*Controls | PBW621*Patients | -0.061 | 0.570 | -0.274 | 0.152 |
| K78*Controls | PBW621*Controls | -0.016 | 0.899 | -0.262 | 0.231 |
| K78*Controls | PHA-P*Patients | 0.049 | 0.648 | -0.164 | 0.262 |
| K78*Controls | PHA-P*Controls | -0.131 | 0.293 | -0.377 | 0.116 |
| K78*Controls | No Ag*Patients | 0.247 | 0.024 | 0.034 | 0.461 |
| K78*Controls | No Ag*Controls | 0.215 | 0.087 | -0.032 | 0.461 |
| C273*Patients | C273*Controls | -0.164 | 0.130 | -0.377 | 0.049 |
| C273*Patients | C591*Patients | -0.185 | 0.037 | -0.359 | -0.011 |
| C273*Patients | C591*Controls | -0.264 | 0.016 | -0.477 | -0.051 |
| C273*Patients | 9D*Patients | -0.162 | 0.067 | -0.337 | 0.012 |
| C273*Patients | 9D*Controls | -0.143 | 0.184 | -0.357 | 0.070 |
| C273*Patients | PBW621*Patients | -0.287 | 0.002 | -0.461 | -0.113 |
| C273*Patients | PBW621*Controls | -0.241 | 0.027 | -0.455 | -0.028 |
| C273*Patients | PHA-P*Patients | -0.177 | 0.047 | -0.351 | -0.002 |
| C273*Patients | PHA-P*Controls | -0.356 | 0.001 | -0.570 | -0.143 |
| C273*Patients | No Ag*Patients | 0.022 | 0.805 | -0.153 | 0.196 |
| C273*Patients | No Ag*Controls | -0.011 | 0.917 | -0.224 | 0.202 |
| C273*Controls | C591*Patients | -0.021 | 0.842 | -0.235 | 0.192 |
| C273*Controls | C591*Controls | -0.100 | 0.421 | -0.346 | 0.146 |
| C273*Controls | 9D*Patients | 0.002 | 0.989 | -0.212 | 0.215 |
| C273*Controls | 9D*Controls | 0.021 | 0.869 | -0.226 | 0.267 |
| C273*Controls | PBW621*Patients | -0.123 | 0.255 | -0.336 | 0.090 |
| C273*Controls | PBW621*Controls | -0.077 | 0.532 | -0.324 | 0.169 |
| C273*Controls | PHA-P*Patients | -0.013 | 0.905 | -0.226 | 0.201 |
| C273*Controls | PHA-P*Controls | -0.192 | 0.124 | -0.439 | 0.054 |
| C273*Controls | No Ag*Patients | 0.186 | 0.087 | -0.028 | 0.399 |
| C273*Controls | No Ag*Controls | 0.153 | 0.220 | -0.094 | 0.399 |
| C591*Patients | C591*Controls | -0.079 | 0.465 | -0.292 | 0.135 |
| C591*Patients | 9D*Patients | 0.023 | 0.794 | -0.151 | 0.197 |
| C591*Patients | 9D*Controls | 0.042 | 0.697 | -0.171 | 0.255 |
| C591*Patients | PBW621*Patients | -0.101 | 0.249 | -0.276 | 0.073 |
| C591*Patients | PBW621*Controls | -0.056 | 0.601 | -0.269 | 0.157 |
| C591*Patients | PHA-P*Patients | 0.009 | 0.922 | -0.166 | 0.183 |
| C591*Patients | PHA-P*Controls | -0.171 | 0.114 | -0.384 | 0.042 |
| C591*Patients | No Ag*Patients | 0.207 | 0.021 | 0.033 | 0.381 |
| C591*Patients | No Ag*Controls | 0.174 | 0.108 | -0.039 | 0.387 |
| C591* Controls | 9D*Patients | 0.101 | 0.346 | -0.112 | 0.315 |
| C591* Controls | 9D*Controls | 0.120 | 0.333 | -0.126 | 0.367 |
| C591* Controls | PBW621*Patients | -0.023 | 0.831 | -0.236 | 0.190 |
| C591* Controls | PBW621*Controls | 0.022 | 0.856 | -0.224 | 0.269 |
| C591* Controls | PHA-P*Patients | 0.087 | 0.417 | -0.126 | 0.301 |
| C591* Controls | PHA-P*Controls | -0.093 | 0.456 | -0.339 | 0.154 |
| C591* Controls | No Ag*Patients | 0.285 | 0.009 | 0.072 | 0.499 |
| C591* Controls | No Ag*Controls | 0.253 | 0.044 | 0.006 | 0.499 |
| 9D* Patients | 9D*Controls | 0.019 | 0.860 | -0.194 | 0.232 |
| 9D* Patients | PBW621*Patients | -0.124 | 0.159 | -0.299 | 0.050 |
| 9D* Patients | PBW621*Controls | -0.079 | 0.463 | -0.292 | 0.134 |
| 9D* Patients | PHA-P*Patients | -0.014 | 0.871 | -0.188 | 0.160 |
| 9D* Patients | PHA-P*Controls | -0.194 | 0.074 | -0.407 | 0.019 |
| 9D* Patients | No Ag*Patients | 0.184 | 0.039 | 0.010 | 0.358 |
| 9D* Patients | No Ag*Controls | 0.151 | 0.162 | -0.062 | 0.365 |
| 9D* Controls | PBW621*Patients | -0.143 | 0.184 | -0.357 | 0.070 |
| 9D* Controls | PBW621*Controls | -0.098 | 0.430 | -0.344 | 0.148 |
| 9D* Controls | PHA-P*Patients | -0.033 | 0.757 | -0.247 | 0.180 |
| 9D* Controls | PHA-P*Controls | -0.213 | 0.089 | -0.459 | 0.033 |
| 9D* Controls | No Ag*Patients | 0.165 | 0.127 | -0.048 | 0.378 |
| 9D* Controls | No Ag*Controls | 0.132 | 0.288 | -0.114 | 0.379 |
| PBW-621* Patients | PBW621*Controls | 0.045 | 0.673 | -0.168 | 0.259 |
| PBW-621* Patients | PHA-P*Patients | 0.110 | 0.212 | -0.064 | 0.284 |
| PBW-621* Patients | PHA-P*Controls | -0.070 | 0.517 | -0.283 | 0.144 |
| PBW-621* Patients | No Ag*Patients | 0.308 | 0.001 | 0.134 | 0.483 |
| PBW-621* Patients | No Ag*Controls | 0.276 | 0.012 | 0.062 | 0.489 |
| PBW-621* Controls | PHA-P*Patients | 0.065 | 0.547 | -0.149 | 0.278 |
| PBW-621* Controls | PHA-P*Controls | -0.115 | 0.355 | -0.361 | 0.131 |
| PBW-621* Controls | No Ag*Patients | 0.263 | 0.016 | 0.050 | 0.476 |
| PBW-621* Controls | No Ag*Controls | 0.230 | 0.067 | -0.016 | 0.477 |
| PHA-P* Patients | PHA-P*Controls | -0.180 | 0.097 | -0.393 | 0.034 |
| PHA-P* Patients | No Ag*Patients | 0.198 | 0.026 | 0.024 | 0.372 |
| PHA-P* Patients | No Ag*Controls | 0.165 | 0.126 | -0.048 | 0.379 |
| PHA-P* Controls | No Ag*Patients | 0.378 | 0.001 | 0.165 | 0.591 |
| PHA-P* Controls | No Ag*Controls | 0.345 | 0.007 | 0.099 | 0.592 |
| No Ag* Patients | No Ag*Controls | -0.033 | 0.760 | -0.246 | 0.181 |

1. BRDU-CELL PROLIFERATION (PBMC’s)

| **Fisher's Least-Significant-Difference Test** | | | | | |
| --- | --- | --- | --- | --- | --- |
| **ANTIGEN(i)*EXP_- GR(i)** | **ANTIGEN(j)*EXP_- GR(j)** | **Difference** | **p-Value** | **95% Confidence Interval** | |
|  |  |  |  | **Lower** | **Upper** |
| K78*Patients | K78*Controls | 0.435 | 0.000 | 0.240 | 0.631 |
| K78*Patients | C273*Patients | 0.346 | 0.000 | 0.186 | 0.506 |
| K78*Patients | C273*Controls | 0.378 | 0.000 | 0.183 | 0.574 |
| K78*Patients | C591*Patients | 0.120 | 0.137 | -0.039 | 0.280 |
| K78*Patients | C591*Controls | 0.401 | 0.000 | 0.206 | 0.597 |
| K78*Patients | 9D*Patients | 0.247 | 0.003 | 0.087 | 0.407 |
| K78*Patients | 9D*Controls | 0.376 | 0.000 | 0.180 | 0.571 |
| K78*Patients | PBW621*Patients | 0.211 | 0.010 | 0.051 | 0.371 |
| K78*Patients | PBW621*Controls | 0.367 | 0.000 | 0.171 | 0.562 |
| K78*Patients | PHA-P*Patients | 0.061 | 0.448 | -0.099 | 0.221 |
| K78*Patients | PHA-P*Controls | 0.182 | 0.068 | -0.014 | 0.377 |
| K78*Patients | No Ag*Patients | 0.416 | 0.000 | 0.256 | 0.576 |
| K78*Patients | No Ag*Controls | 0.443 | 0.000 | 0.247 | 0.638 |
| K78*Controls | C273*Patients | -0.089 | 0.365 | -0.285 | 0.106 |
| K78*Controls | C273*Controls | -0.057 | 0.616 | -0.283 | 0.169 |
| K78*Controls | C591*Patients | -0.315 | 0.002 | -0.511 | -0.119 |
| K78*Controls | C591*Controls | -0.034 | 0.763 | -0.260 | 0.192 |
| K78*Controls | 9D*Patients | -0.189 | 0.059 | -0.384 | 0.007 |
| K78*Controls | 9D*Controls | -0.060 | 0.599 | -0.286 | 0.166 |
| K78*Controls | PBW621*Patients | -0.224 | 0.025 | -0.420 | -0.029 |
| K78*Controls | PBW621*Controls | -0.068 | 0.547 | -0.294 | 0.157 |
| K78*Controls | PHA-P*Patients | -0.374 | 0.000 | -0.570 | -0.179 |
| K78*Controls | PHA-P*Controls | -0.254 | 0.028 | -0.480 | -0.028 |
| K78*Controls | No Ag*Patients | -0.020 | 0.843 | -0.215 | 0.176 |
| K78*Controls | No Ag*Controls | 0.007 | 0.949 | -0.219 | 0.233 |
| C273*Patients | C273*Controls | 0.032 | 0.742 | -0.163 | 0.228 |
| C273*Patients | C591*Patients | -0.226 | 0.006 | -0.385 | -0.066 |
| C273*Patients | C591*Controls | 0.055 | 0.576 | -0.140 | 0.251 |
| C273*Patients | 9D*Patients | -0.099 | 0.220 | -0.259 | 0.061 |
| C273*Patients | 9D*Controls | 0.030 | 0.763 | -0.166 | 0.225 |
| C273*Patients | PBW621*Patients | -0.135 | 0.097 | -0.295 | 0.025 |
| C273*Patients | PBW621*Controls | 0.021 | 0.832 | -0.175 | 0.216 |
| C273*Patients | PHA-P*Patients | -0.285 | 0.001 | -0.445 | -0.125 |
| C273*Patients | PHA-P*Controls | -0.164 | 0.098 | -0.360 | 0.031 |
| C273*Patients | No Ag*Patients | 0.070 | 0.386 | -0.090 | 0.230 |
| C273*Patients | No Ag*Controls | 0.097 | 0.328 | -0.099 | 0.292 |
| C273*Controls | C591*Patients | -0.258 | 0.010 | -0.454 | -0.062 |
| C273*Controls | C591*Controls | 0.023 | 0.841 | -0.203 | 0.249 |
| C273*Controls | 9D*Patients | -0.132 | 0.184 | -0.327 | 0.064 |
| C273*Controls | 9D*Controls | -0.003 | 0.981 | -0.229 | 0.223 |
| C273*Controls | PBW621*Patients | -0.167 | 0.093 | -0.363 | 0.028 |
| C273*Controls | PBW621*Controls | -0.011 | 0.919 | -0.237 | 0.214 |
| C273*Controls | PHA-P*Patients | -0.317 | 0.002 | -0.513 | -0.122 |
| C273*Controls | PHA-P*Controls | -0.197 | 0.087 | -0.423 | 0.029 |
| C273*Controls | No Ag*Patients | 0.037 | 0.703 | -0.158 | 0.233 |
| C273*Controls | No Ag*Controls | 0.064 | 0.572 | -0.162 | 0.290 |
| C591*Patients | C591*Controls | 0.281 | 0.006 | 0.085 | 0.476 |
| C591*Patients | 9D*Patients | 0.127 | 0.119 | -0.033 | 0.286 |
| C591*Patients | 9D*Controls | 0.255 | 0.011 | 0.060 | 0.451 |
| C591*Patients | PBW621*Patients | 0.091 | 0.261 | -0.069 | 0.250 |
| C591*Patients | PBW621*Controls | 0.247 | 0.014 | 0.051 | 0.442 |
| C591*Patients | PHA-P*Patients | -0.059 | 0.462 | -0.219 | 0.100 |
| C591*Patients | PHA-P*Controls | 0.061 | 0.534 | -0.134 | 0.257 |
| C591*Patients | No Ag*Patients | 0.296 | 0.000 | 0.136 | 0.455 |
| C591*Patients | No Ag*Controls | 0.322 | 0.002 | 0.127 | 0.518 |
| C591* Controls | 9D*Patients | -0.154 | 0.120 | -0.350 | 0.041 |
| C591* Controls | 9D*Controls | -0.025 | 0.822 | -0.251 | 0.200 |
| C591* Controls | PBW621*Patients | -0.190 | 0.057 | -0.386 | 0.006 |
| C591* Controls | PBW621*Controls | -0.034 | 0.763 | -0.260 | 0.192 |
| C591* Controls | PHA-P*Patients | -0.340 | 0.001 | -0.536 | -0.144 |
| C591* Controls | PHA-P*Controls | -0.219 | 0.057 | -0.445 | 0.006 |
| C591* Controls | No Ag*Patients | 0.015 | 0.881 | -0.181 | 0.210 |
| C591* Controls | No Ag*Controls | 0.042 | 0.715 | -0.184 | 0.267 |
| 9D* Patients | 9D*Controls | 0.129 | 0.194 | -0.067 | 0.324 |
| 9D* Patients | PBW621*Patients | -0.036 | 0.657 | -0.195 | 0.124 |
| 9D* Patients | PBW621*Controls | 0.120 | 0.225 | -0.076 | 0.316 |
| 9D* Patients | PHA-P*Patients | -0.186 | 0.023 | -0.345 | -0.026 |
| 9D* Patients | PHA-P*Controls | -0.065 | 0.508 | -0.261 | 0.130 |
| 9D* Patients | No Ag*Patients | 0.169 | 0.038 | 0.009 | 0.329 |
| 9D* Patients | No Ag*Controls | 0.196 | 0.050 | 0.000 | 0.391 |
| 9D* Controls | PBW621*Patients | -0.165 | 0.098 | -0.360 | 0.031 |
| 9D* Controls | PBW621*Controls | -0.009 | 0.939 | -0.235 | 0.217 |
| 9D* Controls | PHA-P*Patients | -0.315 | 0.002 | -0.510 | -0.119 |
| 9D* Controls | PHA-P*Controls | -0.194 | 0.091 | -0.420 | 0.032 |
| 9D* Controls | No Ag*Patients | 0.040 | 0.683 | -0.155 | 0.236 |
| 9D* Controls | No Ag*Controls | 0.067 | 0.556 | -0.159 | 0.293 |
| PBW-621* Patients | PBW621*Controls | 0.156 | 0.117 | -0.040 | 0.351 |
| PBW-621* Patients | PHA-P*Patients | -0.150 | 0.065 | -0.310 | 0.010 |
| PBW-621* Patients | PHA-P*Controls | -0.029 | 0.764 | -0.225 | 0.166 |
| PBW-621* Patients | No Ag*Patients | 0.205 | 0.013 | 0.045 | 0.364 |
| PBW-621* Patients | No Ag*Controls | 0.232 | 0.021 | 0.036 | 0.427 |
| PBW-621* Controls | PHA-P*Patients | -0.306 | 0.003 | -0.501 | -0.110 |
| PBW-621* Controls | PHA-P*Controls | -0.185 | 0.106 | -0.411 | 0.041 |
| PBW-621* Controls | No Ag*Patients | 0.049 | 0.619 | -0.147 | 0.245 |
| PBW-621* Controls | No Ag*Controls | 0.076 | 0.506 | -0.150 | 0.302 |
| PHA-P* Patients | PHA-P*Controls | 0.121 | 0.223 | -0.075 | 0.316 |
| PHA-P* Patients | No Ag*Patients | 0.355 | 0.000 | 0.195 | 0.514 |
| PHA-P* Patients | No Ag*Controls | 0.382 | 0.000 | 0.186 | 0.577 |
| PHA-P* Controls | No Ag*Patients | 0.234 | 0.020 | 0.039 | 0.430 |
| PHA-P* Controls | No Ag*Controls | 0.261 | 0.024 | 0.035 | 0.487 |
| No Ag* Patients | No Ag*Controls | 0.027 | 0.786 | -0.169 | 0.222 |

Table 1 (supp) ANOVA (Posthoc test). Least significant difference of celiac disease patient cells and non- celiac controls cultured with wheat antigen from different varieties and compared among each other. (BrdU cell proliferative interaction*) A) Biopsy B) PBMC’s.

*Proliferative interactions – Rate of cell proliferation of biopsy derived T-cells and PBMC’s of celiac disease patients supplemented with wheat antigen from variety C273 compared to T-cells and PBMC’s of celiac disease patients and non-CD controls cultured with wheat antigen from other test varieties and PHA-P.*

1. IFN-γ Production (Biopsy)

| **Fisher's Least-Significant-Difference Test** | | | | | |
| --- | --- | --- | --- | --- | --- |
| **ANTIGEN(i)*EXP_- GR(i)** | **ANTIGEN(j)*EXP_- GR(j)** | **Difference** | **p-Value** | **95% Confidence Interval** | |
|  |  |  |  | **Lower** | **Upper** |
| K78*Patients | K78*Controls | 11.688 | 0.058 | -0.389 | 23.764 |
| K78*Patients | C273*Patients | 16.458 | 0.001 | 6.598 | 26.319 |
| K78*Patients | C273*Controls | 24.646 | 0.000 | 12.569 | 36.722 |
| K78*Patients | C591*Patients | -5.750 | 0.249 | -15.610 | 4.110 |
| K78*Patients | C591*Controls | 20.438 | 0.001 | 8.361 | 32.514 |
| K78*Patients | 9D*Patients | 9.688 | 0.054 | -0.173 | 19.548 |
| K78*Patients | 9D*Controls | 23.146 | 0.000 | 11.069 | 35.222 |
| K78*Patients | PBW621*Patients | 4.792 | 0.336 | -5.069 | 14.652 |
| K78*Patients | PBW621*Controls | 22.229 | 0.000 | 10.153 | 34.306 |
| K78*Patients | PHA-P*Patients | -7.646 | 0.126 | -17.506 | 2.215 |
| K78*Patients | PHA-P*Controls | 15.021 | 0.016 | 2.944 | 27.097 |
| K78*Patients | No Ag*Patients | 23.146 | 0.000 | 13.285 | 33.006 |
| K78*Patients | No Ag*Controls | 1.271 | 0.834 | -10.806 | 13.347 |
| K78*Controls | C273*Patients | 4.771 | 0.433 | -7.306 | 16.847 |
| K78*Controls | C273*Controls | 12.958 | 0.068 | -0.986 | 26.903 |
| K78*Controls | C591*Patients | -17.438 | 0.005 | -29.514 | -5.361 |
| K78*Controls | C591*Controls | 8.750 | 0.215 | -5.195 | 22.695 |
| K78*Controls | 9D*Patients | -2.000 | 0.742 | -14.077 | 10.077 |
| K78*Controls | 9D*Controls | 11.458 | 0.106 | -2.486 | 25.403 |
| K78*Controls | PBW621*Patients | -6.896 | 0.259 | -18.972 | 5.181 |
| K78*Controls | PBW621*Controls | 10.542 | 0.136 | -3.403 | 24.486 |
| K78*Controls | PHA-P*Patients | -19.333 | 0.002 | -31.410 | -7.257 |
| K78*Controls | PHA-P*Controls | 3.333 | 0.635 | -10.611 | 17.278 |
| K78*Controls | No Ag*Patients | 11.458 | 0.063 | -0.618 | 23.535 |
| K78*Controls | No Ag*Controls | -10.417 | 0.141 | -24.361 | 3.528 |
| C273*Patients | C273*Controls | 8.188 | 0.181 | -3.889 | 20.264 |
| C273*Patients | C591*Patients | -22.208 | 0.000 | -32.069 | -12.348 |
| C273*Patients | C591*Controls | 3.979 | 0.513 | -8.097 | 16.056 |
| C273*Patients | 9D*Patients | -6.771 | 0.175 | -16.631 | 3.090 |
| C273*Patients | 9D*Controls | 6.688 | 0.273 | -5.389 | 18.764 |
| C273*Patients | PBW621*Patients | -11.667 | 0.021 | -21.527 | -1.806 |
| C273*Patients | PBW621*Controls | 5.771 | 0.344 | -6.306 | 17.847 |
| C273*Patients | PHA-P*Patients | -24.104 | 0.000 | -33.965 | -14.244 |
| C273*Patients | PHA-P*Controls | -1.438 | 0.813 | -13.514 | 10.639 |
| C273*Patients | No Ag*Patients | 6.687 | 0.181 | -3.173 | 16.548 |
| C273*Patients | No Ag*Controls | -15.187 | 0.014 | -27.264 | -3.111 |
| C273*Controls | C591*Patients | -30.396 | 0.000 | -42.472 | -18.319 |
| C273*Controls | C591*Controls | -4.208 | 0.549 | -18.153 | 9.736 |
| C273*Controls | 9D*Patients | -14.958 | 0.016 | -27.035 | -2.882 |
| C273*Controls | 9D*Controls | -1.500 | 0.831 | -15.445 | 12.445 |
| C273*Controls | PBW621*Patients | -19.854 | 0.002 | -31.931 | -7.778 |
| C273*Controls | PBW621*Controls | -2.417 | 0.731 | -16.361 | 11.528 |
| C273*Controls | PHA-P*Patients | -32.292 | 0.000 | -44.368 | -20.215 |
| C273*Controls | PHA-P*Controls | -9.625 | 0.173 | -23.570 | 4.320 |
| C273*Controls | No Ag*Patients | -1.500 | 0.805 | -13.577 | 10.577 |
| C273*Controls | No Ag*Controls | -23.375 | 0.001 | -37.320 | -9.430 |
| C591*Patients | C591*Controls | 26.188 | 0.000 | 14.111 | 38.264 |
| C591*Patients | 9D*Patients | 15.438 | 0.003 | 5.577 | 25.298 |
| C591*Patients | 9D*Controls | 28.896 | 0.000 | 16.819 | 40.972 |
| C591*Patients | PBW621*Patients | 10.542 | 0.036 | 0.681 | 20.402 |
| C591*Patients | PBW621*Controls | 27.979 | 0.000 | 15.903 | 40.056 |
| C591*Patients | PHA-P*Patients | -1.896 | 0.703 | -11.756 | 7.965 |
| C591*Patients | PHA-P*Controls | 20.771 | 0.001 | 8.694 | 32.847 |
| C591*Patients | No Ag*Patients | 28.896 | 0.000 | 19.035 | 38.756 |
| C591*Patients | No Ag*Controls | 7.021 | 0.250 | -5.056 | 19.097 |
| C591* Controls | 9D*Patients | -10.750 | 0.080 | -22.827 | 1.327 |
| C591* Controls | 9D*Controls | 2.708 | 0.700 | -11.236 | 16.653 |
| C591* Controls | PBW621*Patients | -15.646 | 0.012 | -27.722 | -3.569 |
| C591* Controls | PBW621*Controls | 1.792 | 0.799 | -12.153 | 15.736 |
| C591* Controls | PHA-P*Patients | -28.083 | 0.000 | -40.160 | -16.007 |
| C591* Controls | PHA-P*Controls | -5.417 | 0.441 | -19.361 | 8.528 |
| C591* Controls | No Ag*Patients | 2.708 | 0.656 | -9.368 | 14.785 |
| C591* Controls | No Ag*Controls | -19.167 | 0.008 | -33.111 | -5.222 |
| 9D* Patients | 9D*Controls | 13.458 | 0.029 | 1.382 | 25.535 |
| 9D* Patients | PBW621*Patients | -4.896 | 0.325 | -14.756 | 4.965 |
| 9D* Patients | PBW621*Controls | 12.542 | 0.042 | 0.465 | 24.618 |
| 9D* Patients | PHA-P*Patients | -17.333 | 0.001 | -27.194 | -7.473 |
| 9D* Patients | PHA-P*Controls | 5.333 | 0.381 | -6.743 | 17.410 |
| 9D* Patients | No Ag*Patients | 13.458 | 0.008 | 3.598 | 23.319 |
| 9D* Patients | No Ag*Controls | -8.417 | 0.169 | -20.493 | 3.660 |
| 9D* Controls | PBW621*Patients | -18.354 | 0.003 | -30.431 | -6.278 |
| 9D* Controls | PBW621*Controls | -0.917 | 0.896 | -14.861 | 13.028 |
| 9D* Controls | PHA-P*Patients | -30.792 | 0.000 | -42.868 | -18.715 |
| 9D* Controls | PHA-P*Controls | -8.125 | 0.249 | -22.070 | 5.820 |
| 9D* Controls | No Ag*Patients | 0.000 | 1.000 | -12.077 | 12.077 |
| 9D* Controls | No Ag*Controls | -21.875 | 0.003 | -35.820 | -7.930 |
| PBW-621* Patients | PBW621*Controls | 17.438 | 0.005 | 5.361 | 29.514 |
| PBW-621* Patients | PHA-P*Patients | -12.438 | 0.014 | -22.298 | -2.577 |
| PBW-621* Patients | PHA-P*Controls | 10.229 | 0.096 | -1.847 | 22.306 |
| PBW-621* Patients | No Ag*Patients | 18.354 | 0.000 | 8.494 | 28.215 |
| PBW-621* Patients | No Ag*Controls | -3.521 | 0.563 | -15.597 | 8.556 |
| PBW-621* Controls | PHA-P*Patients | -29.875 | 0.000 | -41.952 | -17.798 |
| PBW-621* Controls | PHA-P*Controls | -7.208 | 0.306 | -21.153 | 6.736 |
| PBW-621* Controls | No Ag*Patients | 0.917 | 0.880 | -11.160 | 12.993 |
| PBW-621* Controls | No Ag*Controls | -20.958 | 0.004 | -34.903 | -7.014 |
| PHA-P* Patients | PHA-P*Controls | 22.667 | 0.000 | 10.590 | 34.743 |
| PHA-P* Patients | No Ag*Patients | 30.792 | 0.000 | 20.931 | 40.652 |
| PHA-P* Patients | No Ag*Controls | 8.917 | 0.145 | -3.160 | 20.993 |
| PHA-P* Controls | No Ag*Patients | 8.125 | 0.184 | -3.952 | 20.202 |
| PHA-P* Controls | No Ag*Controls | -13.750 | 0.053 | -27.695 | 0.195 |
| No Ag* Patients | No Ag*Controls | -21.875 | 0.001 | -33.952 | -9.798 |

1. IFN-γ Production (PBMC’s)

| **Fisher's Least-Significant-Difference Test** | | | | | |
| --- | --- | --- | --- | --- | --- |
| **ANTIGEN(i)*EXP_- GR(i)** | **ANTIGEN(j)*EXP_- GR(j)** | **Difference** | **p-Value** | **95% Confidence Interval** | |
|  |  |  |  | **Lower** | **Upper** |
| K78*Patients | K78*Controls | 21.271 | 0.067 | -1.535 | 44.077 |
| K78*Patients | C273*Patients | 9.500 | 0.312 | -9.121 | 28.121 |
| K78*Patients | C273*Controls | 27.729 | 0.018 | 4.923 | 50.535 |
| K78*Patients | C591*Patients | -17.854 | 0.060 | -36.475 | 0.767 |
| K78*Patients | C591*Controls | 25.646 | 0.028 | 2.840 | 48.452 |
| K78*Patients | 9D*Patients | -17.583 | 0.064 | -36.205 | 1.038 |
| K78*Patients | 9D*Controls | 26.479 | 0.024 | 3.673 | 49.285 |
| K78*Patients | PBW621*Patients | 11.750 | 0.212 | -6.871 | 30.371 |
| K78*Patients | PBW621*Controls | 24.396 | 0.036 | 1.590 | 47.202 |
| K78*Patients | PHA-P*Patients | -20.292 | 0.033 | -38.913 | -1.670 |
| K78*Patients | PHA-P*Controls | 24.354 | 0.037 | 1.548 | 47.160 |
| K78*Patients | No Ag*Patients | 13.333 | 0.158 | -5.288 | 31.955 |
| K78*Patients | No Ag*Controls | 15.437 | 0.181 | -7.369 | 38.244 |
| K78*Controls | C273*Patients | -11.771 | 0.307 | -34.577 | 11.035 |
| K78*Controls | C273*Controls | 6.458 | 0.626 | -19.876 | 32.793 |
| K78*Controls | C591*Patients | -39.125 | 0.001 | -61.931 | -16.319 |
| K78*Controls | C591*Controls | 4.375 | 0.741 | -21.959 | 30.709 |
| K78*Controls | 9D*Patients | -38.854 | 0.001 | -61.660 | -16.048 |
| K78*Controls | 9D*Controls | 5.208 | 0.694 | -21.126 | 31.543 |
| K78*Controls | PBW621*Patients | -9.521 | 0.408 | -32.327 | 13.285 |
| K78*Controls | PBW621*Controls | 3.125 | 0.814 | -23.209 | 29.459 |
| K78*Controls | PHA-P*Patients | -41.562 | 0.001 | -64.369 | -18.756 |
| K78*Controls | PHA-P*Controls | 3.083 | 0.816 | -23.251 | 29.418 |
| K78*Controls | No Ag*Patients | -7.937 | 0.490 | -30.744 | 14.869 |
| K78*Controls | No Ag*Controls | -5.833 | 0.660 | -32.168 | 20.501 |
| C273*Patients | C273*Controls | 18.229 | 0.115 | -4.577 | 41.035 |
| C273*Patients | C591*Patients | -27.354 | 0.005 | -45.975 | -8.733 |
| C273*Patients | C591*Controls | 16.146 | 0.162 | -6.660 | 38.952 |
| C273*Patients | 9D*Patients | -27.083 | 0.005 | -45.705 | -8.462 |
| C273*Patients | 9D*Controls | 16.979 | 0.142 | -5.827 | 39.785 |
| C273*Patients | PBW621*Patients | 2.250 | 0.810 | -16.371 | 20.871 |
| C273*Patients | PBW621*Controls | 14.896 | 0.197 | -7.910 | 37.702 |
| C273*Patients | PHA-P*Patients | -29.792 | 0.002 | -48.413 | -11.170 |
| C273*Patients | PHA-P*Controls | 14.854 | 0.198 | -7.952 | 37.660 |
| C273*Patients | No Ag*Patients | 3.833 | 0.683 | -14.788 | 22.455 |
| C273*Patients | No Ag*Controls | 5.937 | 0.605 | -16.869 | 28.744 |
| C273*Controls | C591*Patients | -45.583 | 0.000 | -68.390 | -22.777 |
| C273*Controls | C591*Controls | -2.083 | 0.875 | -28.418 | 24.251 |
| C273*Controls | 9D*Patients | -45.312 | 0.000 | -68.119 | -22.506 |
| C273*Controls | 9D*Controls | -1.250 | 0.925 | -27.584 | 25.084 |
| C273*Controls | PBW621*Patients | -15.979 | 0.167 | -38.785 | 6.827 |
| C273*Controls | PBW621*Controls | -3.333 | 0.801 | -29.668 | 23.001 |
| C273*Controls | PHA-P*Patients | -48.021 | 0.000 | -70.827 | -25.215 |
| C273*Controls | PHA-P*Controls | -3.375 | 0.799 | -29.709 | 22.959 |
| C273*Controls | No Ag*Patients | -14.396 | 0.212 | -37.202 | 8.410 |
| C273*Controls | No Ag*Controls | -12.292 | 0.355 | -38.626 | 14.043 |
| C591*Patients | C591*Controls | 43.500 | 0.000 | 20.694 | 66.306 |
| C591*Patients | 9D*Patients | 0.271 | 0.977 | -18.350 | 18.892 |
| C591*Patients | 9D*Controls | 44.333 | 0.000 | 21.527 | 67.140 |
| C591*Patients | PBW621*Patients | 29.604 | 0.002 | 10.983 | 48.225 |
| C591*Patients | PBW621*Controls | 42.250 | 0.000 | 19.444 | 65.056 |
| C591*Patients | PHA-P*Patients | -2.437 | 0.795 | -21.059 | 16.184 |
| C591*Patients | PHA-P*Controls | 42.208 | 0.000 | 19.402 | 65.015 |
| C591*Patients | No Ag*Patients | 31.188 | 0.001 | 12.566 | 49.809 |
| C591*Patients | No Ag*Controls | 33.292 | 0.005 | 10.485 | 56.098 |
| C591* Controls | 9D*Patients | -43.229 | 0.000 | -66.035 | -20.423 |
| C591* Controls | 9D*Controls | 0.833 | 0.950 | -25.501 | 27.168 |
| C591* Controls | PBW621*Patients | -13.896 | 0.228 | -36.702 | 8.910 |
| C591* Controls | PBW621*Controls | -1.250 | 0.925 | -27.584 | 25.084 |
| C591* Controls | PHA-P*Patients | -45.937 | 0.000 | -68.744 | -23.131 |
| C591* Controls | PHA-P*Controls | -1.292 | 0.922 | -27.626 | 25.043 |
| C591* Controls | No Ag*Patients | -12.312 | 0.285 | -35.119 | 10.494 |
| C591* Controls | No Ag*Controls | -10.208 | 0.442 | -36.543 | 16.126 |
| 9D* Patients | 9D*Controls | 44.062 | 0.000 | 21.256 | 66.869 |
| 9D* Patients | PBW621*Patients | 29.333 | 0.002 | 10.712 | 47.955 |
| 9D* Patients | PBW621*Controls | 41.979 | 0.000 | 19.173 | 64.785 |
| 9D* Patients | PHA-P*Patients | -2.708 | 0.773 | -21.330 | 15.913 |
| 9D* Patients | PHA-P*Controls | 41.937 | 0.000 | 19.131 | 64.744 |
| 9D* Patients | No Ag*Patients | 30.917 | 0.001 | 12.295 | 49.538 |
| 9D* Patients | No Ag*Controls | 33.021 | 0.005 | 10.215 | 55.827 |
| 9D* Controls | PBW621*Patients | -14.729 | 0.202 | -37.535 | 8.077 |
| 9D* Controls | PBW621*Controls | -2.083 | 0.875 | -28.418 | 24.251 |
| 9D* Controls | PHA-P*Patients | -46.771 | 0.000 | -69.577 | -23.965 |
| 9D* Controls | PHA-P*Controls | -2.125 | 0.873 | -28.459 | 24.209 |
| 9D* Controls | No Ag*Patients | -13.146 | 0.254 | -35.952 | 9.660 |
| 9D* Controls | No Ag*Controls | -11.042 | 0.406 | -37.376 | 15.293 |
| PBW-621* Patients | PBW621*Controls | 12.646 | 0.273 | -10.160 | 35.452 |
| PBW-621* Patients | PHA-P*Patients | -32.042 | 0.001 | -50.663 | -13.420 |
| PBW-621* Patients | PHA-P*Controls | 12.604 | 0.274 | -10.202 | 35.410 |
| PBW-621* Patients | No Ag*Patients | 1.583 | 0.866 | -17.038 | 20.205 |
| PBW-621* Patients | No Ag*Controls | 3.687 | 0.748 | -19.119 | 26.494 |
| PBW-621* Controls | PHA-P*Patients | -44.687 | 0.000 | -67.494 | -21.881 |
| PBW-621* Controls | PHA-P*Controls | -0.042 | 0.997 | -26.376 | 26.293 |
| PBW-621* Controls | No Ag*Patients | -11.062 | 0.337 | -33.869 | 11.744 |
| PBW-621* Controls | No Ag*Controls | -8.958 | 0.500 | -35.293 | 17.376 |
| PHA-P* Patients | PHA-P*Controls | 44.646 | 0.000 | 21.840 | 67.452 |
| PHA-P* Patients | No Ag*Patients | 33.625 | 0.001 | 15.004 | 52.246 |
| PHA-P* Patients | No Ag*Controls | 35.729 | 0.003 | 12.923 | 58.535 |
| PHA-P* Controls | No Ag*Patients | -11.021 | 0.338 | -33.827 | 11.785 |
| PHA-P* Controls | No Ag*Controls | -8.917 | 0.502 | -35.251 | 17.418 |
| No Ag* Patients | No Ag*Controls | 2.104 | 0.855 | -20.702 | 24.910 |

Table 2 (supp) ANOVA (Posthoc test) - Least significant difference of celiac disease patient cells and non- celiac controls cultured with wheat antigen from different varieties and compared among each other. (*IFN-γ* production) A) Biopsy B) PBMC’s.

1. TNF – α Production (Biopsy)

| **Fisher's Least-Significant-Difference Test** | | | | | |
| --- | --- | --- | --- | --- | --- |
| **ANTIGEN(i)*EXP_- GR(i)** | **ANTIGEN(j)*EXP_- GR(j)** | **Difference** | **p-Value** | **95% Confidence Interval** | |
|  |  |  |  | **Lower** | **Upper** |
| K78*Patients | K78*Controls | 45.438 | 0.014 | 9.295 | 81.580 |
| K78*Patients | C273*Patients | 69.000 | 0.000 | 39.490 | 98.510 |
| K78*Patients | C273*Controls | 55.438 | 0.003 | 19.295 | 91.580 |
| K78*Patients | C591*Patients | 11.313 | 0.447 | -18.198 | 40.823 |
| K78*Patients | C591*Controls | -15.187 | 0.405 | -51.330 | 20.955 |
| K78*Patients | 9D*Patients | -8.375 | 0.573 | -37.885 | 21.135 |
| K78*Patients | 9D*Controls | -7.062 | 0.698 | -43.205 | 29.080 |
| K78*Patients | PBW621*Patients | -12.938 | 0.385 | -42.448 | 16.573 |
| K78*Patients | PBW621*Controls | 15.438 | 0.397 | -20.705 | 51.580 |
| K78*Patients | PHA-P*Patients | 38.563 | 0.011 | 9.052 | 68.073 |
| K78*Patients | PHA-P*Controls | 47.938 | 0.010 | 11.795 | 84.080 |
| K78*Patients | No Ag*Patients | 74.438 | 0.000 | 44.927 | 103.948 |
| K78*Patients | No Ag*Controls | 56.688 | 0.003 | 20.545 | 92.830 |
| K78*Controls | C273*Patients | 23.562 | 0.198 | -12.580 | 59.705 |
| K78*Controls | C273*Controls | 10.000 | 0.634 | -31.733 | 51.733 |
| K78*Controls | C591*Patients | -34.125 | 0.064 | -70.267 | 2.017 |
| K78*Controls | C591*Controls | -60.625 | 0.005 | -102.358 | -18.892 |
| K78*Controls | 9D*Patients | -53.813 | 0.004 | -89.955 | -17.670 |
| K78*Controls | 9D*Controls | -52.500 | 0.014 | -94.233 | -10.767 |
| K78*Controls | PBW621*Patients | -58.375 | 0.002 | -94.517 | -22.233 |
| K78*Controls | PBW621*Controls | -30.000 | 0.156 | -71.733 | 11.733 |
| K78*Controls | PHA-P*Patients | -6.875 | 0.706 | -43.017 | 29.267 |
| K78*Controls | PHA-P*Controls | 2.500 | 0.905 | -39.233 | 44.233 |
| K78*Controls | No Ag*Patients | 29.000 | 0.114 | -7.142 | 65.142 |
| K78*Controls | No Ag*Controls | 11.250 | 0.593 | -30.483 | 52.983 |
| C273*Patients | C273*Controls | -13.562 | 0.457 | -49.705 | 22.580 |
| C273*Patients | C591*Patients | -57.688 | 0.000 | -87.198 | -28.177 |
| C273*Patients | C591*Controls | -84.187 | 0.000 | -120.330 | -48.045 |
| C273*Patients | 9D*Patients | -77.375 | 0.000 | -106.885 | -47.865 |
| C273*Patients | 9D*Controls | -76.062 | 0.000 | -112.205 | -39.920 |
| C273*Patients | PBW621*Patients | -81.938 | 0.000 | -111.448 | -52.427 |
| C273*Patients | PBW621*Controls | -53.562 | 0.004 | -89.705 | -17.420 |
| C273*Patients | PHA-P*Patients | -30.438 | 0.043 | -59.948 | -0.927 |
| C273*Patients | PHA-P*Controls | -21.062 | 0.249 | -57.205 | 15.080 |
| C273*Patients | No Ag*Patients | 5.438 | 0.714 | -24.073 | 34.948 |
| C273*Patients | No Ag*Controls | -12.312 | 0.499 | -48.455 | 23.830 |
| C273*Controls | C591*Patients | -44.125 | 0.017 | -80.267 | -7.983 |
| C273*Controls | C591*Controls | -70.625 | 0.001 | -112.358 | -28.892 |
| C273*Controls | 9D*Patients | -63.813 | 0.001 | -99.955 | -27.670 |
| C273*Controls | 9D*Controls | -62.500 | 0.004 | -104.233 | -20.767 |
| C273*Controls | PBW621*Patients | -68.375 | 0.000 | -104.517 | -32.233 |
| C273*Controls | PBW621*Controls | -40.000 | 0.060 | -81.733 | 1.733 |
| C273*Controls | PHA-P*Patients | -16.875 | 0.355 | -53.017 | 19.267 |
| C273*Controls | PHA-P*Controls | -7.500 | 0.721 | -49.233 | 34.233 |
| C273*Controls | No Ag*Patients | 19.000 | 0.298 | -17.142 | 55.142 |
| C273*Controls | No Ag*Controls | 1.250 | 0.953 | -40.483 | 42.983 |
| C591*Patients | C591*Controls | -26.500 | 0.148 | -62.642 | 9.642 |
| C591*Patients | 9D*Patients | -19.688 | 0.188 | -49.198 | 9.823 |
| C591*Patients | 9D*Controls | -18.375 | 0.314 | -54.517 | 17.767 |
| C591*Patients | PBW621*Patients | -24.250 | 0.106 | -53.760 | 5.260 |
| C591*Patients | PBW621*Controls | 4.125 | 0.821 | -32.017 | 40.267 |
| C591*Patients | PHA-P*Patients | 27.250 | 0.070 | -2.260 | 56.760 |
| C591*Patients | PHA-P*Controls | 36.625 | 0.047 | 0.483 | 72.767 |
| C591*Patients | No Ag*Patients | 63.125 | 0.000 | 33.615 | 92.635 |
| C591*Patients | No Ag*Controls | 45.375 | 0.015 | 9.233 | 81.517 |
| C591* Controls | 9D*Patients | 6.812 | 0.708 | -29.330 | 42.955 |
| C591* Controls | 9D*Controls | 8.125 | 0.699 | -33.608 | 49.858 |
| C591* Controls | PBW621*Patients | 2.250 | 0.902 | -33.892 | 38.392 |
| C591* Controls | PBW621*Controls | 30.625 | 0.148 | -11.108 | 72.358 |
| C591* Controls | PHA-P*Patients | 53.750 | 0.004 | 17.608 | 89.892 |
| C591* Controls | PHA-P*Controls | 63.125 | 0.004 | 21.392 | 104.858 |
| C591* Controls | No Ag*Patients | 89.625 | 0.000 | 53.483 | 125.767 |
| C591* Controls | No Ag*Controls | 71.875 | 0.001 | 30.142 | 113.608 |
| 9D* Patients | 9D*Controls | 1.313 | 0.942 | -34.830 | 37.455 |
| 9D* Patients | PBW621*Patients | -4.563 | 0.759 | -34.073 | 24.948 |
| 9D* Patients | PBW621*Controls | 23.813 | 0.193 | -12.330 | 59.955 |
| 9D* Patients | PHA-P*Patients | 46.938 | 0.002 | 17.427 | 76.448 |
| 9D* Patients | PHA-P*Controls | 56.313 | 0.003 | 20.170 | 92.455 |
| 9D* Patients | No Ag*Patients | 82.813 | 0.000 | 53.302 | 112.323 |
| 9D* Patients | No Ag*Controls | 65.063 | 0.001 | 28.920 | 101.205 |
| 9D* Controls | PBW621*Patients | -5.875 | 0.747 | -42.017 | 30.267 |
| 9D* Controls | PBW621*Controls | 22.500 | 0.286 | -19.233 | 64.233 |
| 9D* Controls | PHA-P*Patients | 45.625 | 0.014 | 9.483 | 81.767 |
| 9D* Controls | PHA-P*Controls | 55.000 | 0.011 | 13.267 | 96.733 |
| 9D* Controls | No Ag*Patients | 81.500 | 0.000 | 45.358 | 117.642 |
| 9D* Controls | No Ag*Controls | 63.750 | 0.003 | 22.017 | 105.483 |
| PBW-621* Patients | PBW621*Controls | 28.375 | 0.122 | -7.767 | 64.517 |
| PBW-621* Patients | PHA-P*Patients | 51.500 | 0.001 | 21.990 | 81.010 |
| PBW-621* Patients | PHA-P*Controls | 60.875 | 0.001 | 24.733 | 97.017 |
| PBW-621* Patients | No Ag*Patients | 87.375 | 0.000 | 57.865 | 116.885 |
| PBW-621* Patients | No Ag*Controls | 69.625 | 0.000 | 33.483 | 105.767 |
| PBW-621* Controls | PHA-P*Patients | 23.125 | 0.206 | -13.017 | 59.267 |
| PBW-621* Controls | PHA-P*Controls | 32.500 | 0.125 | -9.233 | 74.233 |
| PBW-621* Controls | No Ag*Patients | 59.000 | 0.002 | 22.858 | 95.142 |
| PBW-621* Controls | No Ag*Controls | 41.250 | 0.053 | -0.483 | 82.983 |
| PHA-P* Patients | PHA-P*Controls | 9.375 | 0.607 | -26.767 | 45.517 |
| PHA-P* Patients | No Ag*Patients | 35.875 | 0.018 | 6.365 | 65.385 |
| PHA-P* Patients | No Ag*Controls | 18.125 | 0.321 | -18.017 | 54.267 |
| PHA-P* Controls | No Ag*Patients | 26.500 | 0.148 | -9.642 | 62.642 |
| PHA-P* Controls | No Ag*Controls | 8.750 | 0.677 | -32.983 | 50.483 |
| No Ag* Patients | No Ag*Controls | -17.750 | 0.331 | -53.892 | 18.392 |

1. TNF – α Production (PBMC’s)

| **Fisher's Least-Significant-Difference Test** | | | | | |
| --- | --- | --- | --- | --- | --- |
| **ANTIGEN(i)*EXP_- GR(i)** | **ANTIGEN(j)*EXP_- GR(j)** | **Difference** | **p-Value** | **95% Confidence Interval** | |
|  |  |  |  | **Lower** | **Upper** |
| K78*Patients | K78*Controls | 37.375 | 0.068 | -2.769 | 77.519 |
| K78*Patients | C273*Patients | 81.563 | 0.000 | 48.785 | 114.340 |
| K78*Patients | C273*Controls | 31.125 | 0.127 | -9.019 | 71.269 |
| K78*Patients | C591*Patients | -3.563 | 0.829 | -36.340 | 29.215 |
| K78*Patients | C591*Controls | 3.000 | 0.882 | -37.144 | 43.144 |
| K78*Patients | 9D*Patients | 5.438 | 0.742 | -27.340 | 38.215 |
| K78*Patients | 9D*Controls | 30.750 | 0.131 | -9.394 | 70.894 |
| K78*Patients | PBW621*Patients | 21.813 | 0.189 | -10.965 | 54.590 |
| K78*Patients | PBW621*Controls | 8.000 | 0.692 | -32.144 | 48.144 |
| K78*Patients | PHA-P*Patients | 74.625 | 0.000 | 41.848 | 107.402 |
| K78*Patients | PHA-P*Controls | 63.625 | 0.002 | 23.481 | 103.769 |
| K78*Patients | No Ag*Patients | 92.688 | 0.000 | 59.910 | 125.465 |
| K78*Patients | No Ag*Controls | 69.250 | 0.001 | 29.106 | 109.394 |
| K78*Controls | C273*Patients | 44.188 | 0.031 | 4.044 | 84.331 |
| K78*Controls | C273*Controls | -6.250 | 0.789 | -52.604 | 40.104 |
| K78*Controls | C591*Patients | -40.937 | 0.046 | -81.081 | -0.794 |
| K78*Controls | C591*Controls | -34.375 | 0.144 | -80.729 | 11.979 |
| K78*Controls | 9D*Patients | -31.937 | 0.117 | -72.081 | 8.206 |
| K78*Controls | 9D*Controls | -6.625 | 0.776 | -52.979 | 39.729 |
| K78*Controls | PBW621*Patients | -15.562 | 0.442 | -55.706 | 24.581 |
| K78*Controls | PBW621*Controls | -29.375 | 0.210 | -75.729 | 16.979 |
| K78*Controls | PHA-P*Patients | 37.250 | 0.068 | -2.894 | 77.394 |
| K78*Controls | PHA-P*Controls | 26.250 | 0.263 | -20.104 | 72.604 |
| K78*Controls | No Ag*Patients | 55.313 | 0.008 | 15.169 | 95.456 |
| K78*Controls | No Ag*Controls | 31.875 | 0.175 | -14.479 | 78.229 |
| C273*Patients | C273*Controls | -50.438 | 0.015 | -90.581 | -10.294 |
| C273*Patients | C591*Patients | -85.125 | 0.000 | -117.902 | -52.348 |
| C273*Patients | C591*Controls | -78.563 | 0.000 | -118.706 | -38.419 |
| C273*Patients | 9D*Patients | -76.125 | 0.000 | -108.902 | -43.348 |
| C273*Patients | 9D*Controls | -50.813 | 0.014 | -90.956 | -10.669 |
| C273*Patients | PBW621*Patients | -59.750 | 0.001 | -92.527 | -26.973 |
| C273*Patients | PBW621*Controls | -73.563 | 0.000 | -113.706 | -33.419 |
| C273*Patients | PHA-P*Patients | -6.938 | 0.674 | -39.715 | 25.840 |
| C273*Patients | PHA-P*Controls | -17.938 | 0.376 | -58.081 | 22.206 |
| C273*Patients | No Ag*Patients | 11.125 | 0.501 | -21.652 | 43.902 |
| C273*Patients | No Ag*Controls | -12.313 | 0.543 | -52.456 | 27.831 |
| C273*Controls | C591*Patients | -34.687 | 0.089 | -74.831 | 5.456 |
| C273*Controls | C591*Controls | -28.125 | 0.230 | -74.479 | 18.229 |
| C273*Controls | 9D*Patients | -25.687 | 0.206 | -65.831 | 14.456 |
| C273*Controls | 9D*Controls | -0.375 | 0.987 | -46.729 | 45.979 |
| C273*Controls | PBW621*Patients | -9.312 | 0.645 | -49.456 | 30.831 |
| C273*Controls | PBW621*Controls | -23.125 | 0.323 | -69.479 | 23.229 |
| C273*Controls | PHA-P*Patients | 43.500 | 0.034 | 3.356 | 83.644 |
| C273*Controls | PHA-P*Controls | 32.500 | 0.166 | -13.854 | 78.854 |
| C273*Controls | No Ag*Patients | 61.563 | 0.003 | 21.419 | 101.706 |
| C273*Controls | No Ag*Controls | 38.125 | 0.105 | -8.229 | 84.479 |
| C591*Patients | C591*Controls | 6.562 | 0.745 | -33.581 | 46.706 |
| C591*Patients | 9D*Patients | 9.000 | 0.586 | -23.777 | 41.777 |
| C591*Patients | 9D*Controls | 34.312 | 0.093 | -5.831 | 74.456 |
| C591*Patients | PBW621*Patients | 25.375 | 0.127 | -7.402 | 58.152 |
| C591*Patients | PBW621*Controls | 11.562 | 0.568 | -28.581 | 51.706 |
| C591*Patients | PHA-P*Patients | 78.188 | 0.000 | 45.410 | 110.965 |
| C591*Patients | PHA-P*Controls | 67.187 | 0.001 | 27.044 | 107.331 |
| C591*Patients | No Ag*Patients | 96.250 | 0.000 | 63.473 | 129.027 |
| C591*Patients | No Ag*Controls | 72.812 | 0.001 | 32.669 | 112.956 |
| C591* Controls | 9D*Patients | 2.438 | 0.904 | -37.706 | 42.581 |
| C591* Controls | 9D*Controls | 27.750 | 0.237 | -18.604 | 74.104 |
| C591* Controls | PBW621*Patients | 18.813 | 0.353 | -21.331 | 58.956 |
| C591* Controls | PBW621*Controls | 5.000 | 0.830 | -41.354 | 51.354 |
| C591* Controls | PHA-P*Patients | 71.625 | 0.001 | 31.481 | 111.769 |
| C591* Controls | PHA-P*Controls | 60.625 | 0.011 | 14.271 | 106.979 |
| C591* Controls | No Ag*Patients | 89.688 | 0.000 | 49.544 | 129.831 |
| C591* Controls | No Ag*Controls | 66.250 | 0.006 | 19.896 | 112.604 |
| 9D* Patients | 9D*Controls | 25.312 | 0.213 | -14.831 | 65.456 |
| 9D* Patients | PBW621*Patients | 16.375 | 0.322 | -16.402 | 49.152 |
| 9D* Patients | PBW621*Controls | 2.562 | 0.899 | -37.581 | 42.706 |
| 9D* Patients | PHA-P*Patients | 69.188 | 0.000 | 36.410 | 101.965 |
| 9D* Patients | PHA-P*Controls | 58.187 | 0.005 | 18.044 | 98.331 |
| 9D* Patients | No Ag*Patients | 87.250 | 0.000 | 54.473 | 120.027 |
| 9D* Patients | No Ag*Controls | 63.812 | 0.002 | 23.669 | 103.956 |
| 9D* Controls | PBW621*Patients | -8.937 | 0.658 | -49.081 | 31.206 |
| 9D* Controls | PBW621*Controls | -22.750 | 0.331 | -69.104 | 23.604 |
| 9D* Controls | PHA-P*Patients | 43.875 | 0.033 | 3.731 | 84.019 |
| 9D* Controls | PHA-P*Controls | 32.875 | 0.162 | -13.479 | 79.229 |
| 9D* Controls | No Ag*Patients | 61.938 | 0.003 | 21.794 | 102.081 |
| 9D* Controls | No Ag*Controls | 38.500 | 0.102 | -7.854 | 84.854 |
| PBW-621* Patients | PBW621*Controls | -13.813 | 0.495 | -53.956 | 26.331 |
| PBW-621* Patients | PHA-P*Patients | 52.813 | 0.002 | 20.035 | 85.590 |
| PBW-621* Patients | PHA-P*Controls | 41.812 | 0.041 | 1.669 | 81.956 |
| PBW-621* Patients | No Ag*Patients | 70.875 | 0.000 | 38.098 | 103.652 |
| PBW-621* Patients | No Ag*Controls | 47.437 | 0.021 | 7.294 | 87.581 |
| PBW-621* Controls | PHA-P*Patients | 66.625 | 0.001 | 26.481 | 106.769 |
| PBW-621* Controls | PHA-P*Controls | 55.625 | 0.019 | 9.271 | 101.979 |
| PBW-621* Controls | No Ag*Patients | 84.688 | 0.000 | 44.544 | 124.831 |
| PBW-621* Controls | No Ag*Controls | 61.250 | 0.010 | 14.896 | 107.604 |
| PHA-P* Patients | PHA-P*Controls | -11.000 | 0.586 | -51.144 | 29.144 |
| PHA-P* Patients | No Ag*Patients | 18.063 | 0.276 | -14.715 | 50.840 |
| PHA-P* Patients | No Ag*Controls | -5.375 | 0.790 | -45.519 | 34.769 |
| PHA-P* Controls | No Ag*Patients | 29.063 | 0.153 | -11.081 | 69.206 |
| PHA-P* Controls | No Ag*Controls | 5.625 | 0.809 | -40.729 | 51.979 |
| No Ag* Patients | No Ag*Controls | -23.438 | 0.248 | -63.581 | 16.706 |

Table 3 (supp) ANOVA (Posthoc test) - Least significant difference of celiac disease patient cells and non- celiac controls cultured with wheat antigen from different varieties and compared among each other. (*TNF-α* production) A) Biopsy B) PBMC’s.
