# Supplementary figures and images for: Variable Immunogenic Potential of Wheat: Prospective for Selection of Innocuous Varieties for Celiac Disease Patients via in vitro Approach
Source: Front Immunol. 2019 Feb 4;10:84. doi: 10.3389/fimmu.2019.00084 (PMC6371638; doi:10.3389/fimmu.2019.00084)

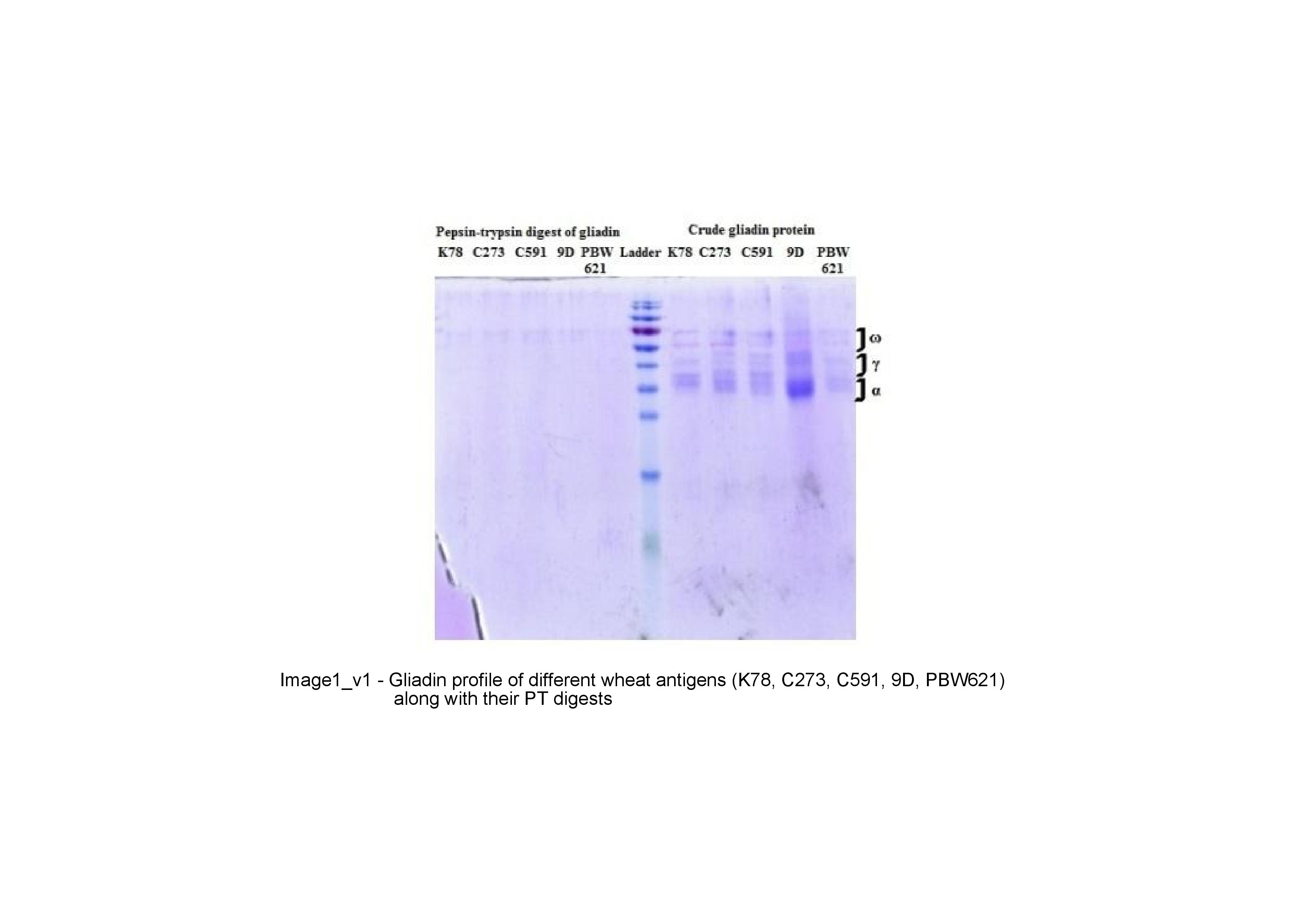

Supplement: Supplementary file 2 [file Image_1.jpg]
